# The right response at the right time: Exploring helminth immune modulation in sticklebacks by experimental coinfection

**DOI:** 10.1111/mec.15106

**Published:** 2019-05-17

**Authors:** Agnes Piecyk, Marc Ritter, Martin Kalbe

**Affiliations:** ^1^ Evolutionary Ecology Max Planck Institute for Evolutionary Biology Plön Germany; ^2^ Marine Evolutionary Ecology GEOMAR Helmholtz Centre for Ocean Research Kiel Kiel Germany

**Keywords:** *Diplostomum pseudospathaceum*, *Gasterosteus aculeatus*, gene expression, helminth immune modulation, host‐parasite interaction, *Schistocephalus solidus*

## Abstract

Parasites are one of the strongest selective agents in nature. They select for hosts that evolve counter‐adaptive strategies to cope with infection. Helminth parasites are special because they can modulate their hosts’ immune responses. This phenomenon is important in epidemiological contexts, where coinfections may be affected. How different types of hosts and helminths interact with each other is insufficiently investigated. We used the three‐spined stickleback (*Gasterosteus aculeatus*) – *Schistocephalus solidus* model to study mechanisms and temporal components of helminth immune modulation. Sticklebacks from two contrasting populations with either high resistance (HR) or low resistance (LR) against *S. solidus,* were individually exposed to *S. solidus* strains with characteristically high growth (HG) or low growth (LG) in *G. aculeatus*. We determined the susceptibility to another parasite, the eye fluke *Diplostomum pseudospathaceum*, and the expression of 23 key immune genes at three time points after *S. solidus* infection. *D. pseudospathaceum* infection rates and the gene expression responses depended on host and *S. solidus* type and changed over time. Whereas the effect of *S. solidus* type was not significant after three weeks, T regulatory responses and complement components were upregulated at later time points if hosts were infected with HG *S. solidus*. HR hosts showed a well orchestrated immune response, which was absent in LR hosts. Our results emphasize the role of regulatory T cells and the timing of specific immune responses during helminth infections. This study elucidates the importance to consider different coevolutionary trajectories and ecologies when studying host‐parasite interactions.

## 1 INTRODUCTION

The evolution of species and species interactions are shaped through a complex web of abiotic and biotic factors (Betts, Rafaluk, & King, 2016; Maizels & Nussey, 2013; Schulenburg, Kurtz, Moret, & Siva‐Jothy, 2009; Sheldon & Verhulst, 1996). One of the key processes is the coevolution between hosts and parasites. Parasites shape the immune function of their host and in response undergo rapid evolution of virulence, which may result in ongoing antagonistic coevolution (Buckling & Rainey, 2002; Dargent, Scott, Hendry, & Fussmann, 2013; Eizaguirre, Lenz, Kalbe, & Milinski, 2012; Paterson et al., 2010). However, the underlying evolutionary trajectories of this coevolution have mostly been studied in species pairs. Such an approach neglects the complexity of natural systems and the consequences of coinfection. Indeed, parasite species can influence one another (Benesh & Kalbe, 2016), especially if multiple parasites infect one host. In such a case, coinfecting parasites interact directly or indirectly, for example through resource competition or effects on host immunity (Betts et al., 2016).

The vertebrate immune system coevolved with helminth parasites (metazoans classified as cestodes, nematodes and trematodes) that are exceptional immune modulators (Anthony, Rutitzky, Urban, Stadecker, & Gause, 2007; Khan & Fallon, 2013; Maizels, 2005). It has been shown that helminth infections can alter susceptibility to macroparasites (Benesh & Kalbe, 2016; Lello, Boag, Fenton, Stevenson, & Hudson, 2004; Pedersen & Antonovics, 2013) and microbes (Giacomin, Croese, Krause, Loukas, & Cantacessi, 2015; Graham, 2008). Moreover, helminth‐mediated downregulation of host immunity is observed to suppress autoimmune or inflammatory disorders such as asthma, rheumatoid arthritis, type 1 diabetes, multiple sclerosis, and inflammatory bowel diseases (Maizels & McSorley, 2016; Maizels & Yazdanbakhsh, 2003).

Helminths typically interfere with characteristic elements of innate and adaptive immunity (Anthony et al., 2007; McSorley, Hewitson, & Maizels, 2013). Most knowledge stems from clinical and experimental work involving human patients or murine systems. A prominent observation is the switch between activities of distinct T helper cell subsets over time. Characteristically, an early T helper 1 (Th1) type response is skewed towards a T helper 2 (Th2) type response in chronic helminth infections. Th1 and Th2 responses are defined by distinct functions and cytokines (Maizels, Bundy, Selkirk, Smith, & Anderson, 1993; Maizels & McSorley, 2016). Th1 type cytokines, such as Interleukin‐1β (IL‐1β) and Tumor necrosis factor α (TNF‐α), are proinflammatory; Th2 type cytokines can inhibit Th1 cells and acute‐phase cytokines, induce alternatively activated macrophages, and stimulate B‐cells and antibody production (Liu, Liu, Bleich, Salgame, & Gause, 2010; Mosmann & Sad, 1996). Nevertheless, high parasite burdens were described despite increased Th2 responses, which brought another T cell subset into focus, namely immunosuppressive regulatory T (Treg) cells (Maizels, 2005; Maizels & McSorley, 2016; Maizels & Yazdanbakhsh, 2003; Nutman, 2015). Tregs are considered to be key controllers of immune system homeostasis and expand upon longstanding helminth infections. Modulation of these cells may protect from immunopathology and ensure the persistence of the parasite within the host. Helminths are also known to interact with the host's complement system (Heath, Holcman, & Shaw, 1994; Mulcahy, O'Neill, Donnelly, & Dalton, 2004) which is considered to link innate and adaptive immunity (Carroll, 2004).

It has recently been suggested that those characteristic elements of innate and adaptive immunity, namely Th1, Th2, Treg cells, and complement components, are of central importance in helminth infections of the three‐spined stickleback *Gasterosteus aculeatus* (hereafter “stickleback”) (Haase et al., 2014, 2016; Robertson, Bradley, & MacColl, 2015). Sticklebacks are widely distributed across the Northern Hemisphere and are naturally infected with a wide diversity of parasites (Feulner et al., 2015; Kalbe, 2002; MacColl, 2009). Parasites seem to drive local adaptation and genomic differentiation in this species (Eizaguirre et al., 2012; Feulner et al., 2015; Robertson et al., 2015). Habitat specific immunity and immune gene expression have been described (Huang et al., 2016; Lenz, Eizaguirre, Rotter, Kalbe, & Milinski, 2013; Lohman, Steinel, Weber, & Bolnick, 2017; Wegner, Reusch, & Kalbe, 2003). However, little is known about temporal changes and the ecological effects of the host's response to infection (see Benesh & Kalbe, 2016; Brunner et al., 2017). In an ecological context, host‐parasite interactions potentially influence the occurrence and ultimately the coevolutionary trajectories of coinfecting parasites and the fitness consequences on the host (Betts et al., 2016).

Here, we used controlled infection experiments with sticklebacks and their specific cestode parasite *Schistocephalus solidus* for a thorough investigation of helminth immune modulation in a model vertebrate system. We tested our predictions by using stickleback and *S. solidus* types with different coevolutionary backgrounds. Our study addressed the ecological significance by exploring the influence on coinfection probability with a naturally co‐occurring parasite, the trematode *Diplostomum pseudospathaceum*. *D. pseudospathaceum* migrates to the immunologically privileged eye lens of the fish within 24 hr and evades adaptive immune responses (Chappell, Hardie, & Secombes, 1994). The potentially inflicted cataract formation within the eyes has the potential to impair *G. aculeatus* predator avoidance (Karvonen, Seppälä, & Valtonen, 2004; Meakins & Walkey, 1975; Seppälä, Karvonen, & Tellervo Valtonen, 2004). Both parasite species have a complex life cycle with *G. aculeatus* as intermediate and piscivorous birds as final hosts. We studied the temporal dynamics by sampling at different time points of *S. solidus* development in the stickleback and determined corresponding host immune gene expression patterns.

*Schistocephalus solidus* has a three‐host life cycle with copepods, *G. aculeatus*, and fish‐eating birds as three consecutive hosts (Barber & Scharsack, 2010; Clarke, 1954; Smyth, 1946). The cestode becomes infective for the final host and is able to reproduce above a weight of approximately 50 mg (Hammerschmidt & Kurtz, 2009; Tierney & Crompton, 1992). This stage has also been reported to mark the onset of *S. solidus* immune modulation that may facilitate the transmission to the final host (Scharsack, Koch, & Hammerschmidt, 2007). *S. solidus* is a common parasite of *G. aculeatus* in freshwater and brackish habitats. The outcome of their coevolution seems to differ greatly between populations (Barber & Scharsack, 2010; Kalbe, Eizaguirre, Scharsack, & Jakobsen, 2016; Weber et al., 2017). While some sticklebacks evolved high resistance against *S. solidus*, measured as the limitation of cestode growth, the resistance of others is less effective (Kalbe et al., 2016; Piecyk, Roth, & Kalbe, 2019; Weber et al., 2017). Likewise, some *S. solidus* types grow consistently fast and reach enormous weights, whereas other strains grow characteristically slow (Benesh & Kalbe, 2016; Kalbe et al., 2016; Piecyk et al., 2019; Ritter, Kalbe, & Henrich, 2017). We chose hosts and parasites from (a) populations with low *S. solidus* prevalence (<1%) and high parasite diversity (Lake Großer Plöner See and Neustädter Binnenwasser, Germany), and (b) a population with high *S. solidus* prevalence (20% to >50%) and low parasite diversity (Lake Skogseidvatnet, Norway) (Table 1). Since immune defence is costly and coevolves with parasite virulence (Duncan, Fellous, & Kaltz, 2011; Sheldon & Verhulst, 1996), it has been proposed that sticklebacks from frequently exposed populations evolved increased resistance and *S. solidus*, being entangled in an arms race of adaptation and counter‐adaptation, evolved increased virulence (Franke et al., 2014; Kalbe et al., 2016; Piecyk et al., 2019; Scharsack et al., 2016). Moreover, high virulence has been reported for populations with low density of nonhost predators ensuring a sufficient transmission rate to the definite hosts of *S. solidus* (Arme & Owen, 1967; Kalbe et al., 2016). It has thus been suggested that the host and parasite types from Germany evolved under de‐escalated arms‐race dynamics causing slow parasite growth (low growth, LG *S. solidus*) and low resistance (LR sticklebacks) and that the host and parasite types from Norway supposedly selected for increased resistance (high resistance, HR sticklebacks) and virulence (high growth, HG *S. solidus*) in their habitat (Kalbe et al., 2016; Piecyk et al., 2019).

**Table 1:** Host and parasite sampling sites.** “**Type” refers to the conceptual resistance and growth types of *G. aculeatus* and *S. solidus*

Type	*Gasterosteus aculeatus*		
LR	Lake “Großer Plöner See”	Germany	54°08'48"N, 10°24'30"E
HR	Lake “Skogseidvatnet”	Norway	60°14'44"N, 5°55'03"E
	*Schistocephalus solidus*		
LG	Lagoon “Neustädter Binnenwasser”	Germany	54°06'40"N, 10°48'50"E
HG	Lake “Skogseidvatnet”	Norway	60°14'44"N, 5°55'03"E
	*Diplostomum pseudospathaceum*		
–	Lake “Kleiner Plöner See” (1)	Germany	54°09'41.6"N 10°22'36.5"E
–	Lake “Kleiner Plöner See” (2)	Germany	54°09'46.2"N 10°24'05.2"E
–	Lake “Bischhofsee”	Germany	54°06'36.7"N 10°25'44.3"E

Abbreviations: HG, high growth; HR, high resistance; LG, low growth; LR, low resistance.

We hypothesized that *S. solidus* modulates immune responses in *G. aculeatus* and that this effect differs between contrasting stickleback and *S. solidus* types, as well as over time. More specifically, we expected modulatory effects when *S. solidus* is able to reproduce upon transmission to the final hosts, which should be earlier in fast growing (HG) than in slow growing (LG) types. We further hypothesized an effective immune response in the coevolved high growth–high resistance (HG‐HR) combination, but not in the unadapted high growth–low resistance (HG‐LR) combination.

**Figure 1:**
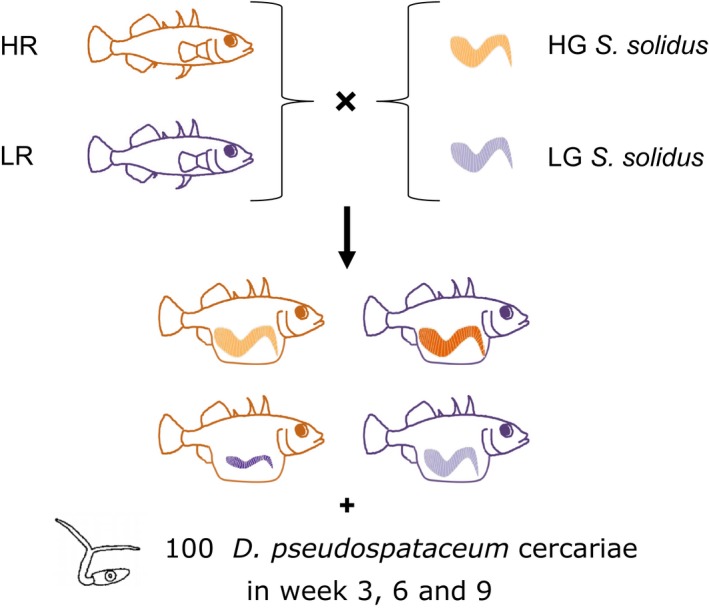
Experimental design. Two stickleback populations of low resistance (LR) and high resistance (HR) were exposed to *Schistocephalus solidus* of high growth (HG) or low growth (LG). Subsets of *S. solidus* exposed sticklebacks were exposed to 100 cercariae of the eye fluke *Diplostomum pseudospathaceum* at distinct time points (after 3, 6 or 9 weeks) [Colour figure can be viewed at http://wileyonlinelibrary.com]

Expression levels of 23 *G. aculeatus* immune genes that may play key roles in *S. solidus* and *D. pseudospathaceum* infection were analyzed to characterize the molecular infection phenotypes. We chose genes that had been identified using transcriptome data (Haase et al., 2014; Huang et al., 2016) and quantitative real‐time PCR studies (Brunner et al., 2017; Robertson et al., 2015; Stutz, Schmerer, Coates, & Bolnick, 2015). Our set includes targets from innate and adaptive immunity as well as complement components. We used subsets of these genes to study Th1, Th2 and Treg responses in further detail. The stickleback's immune system is principally able to eliminate *S. solidus* up to 17 days post infection, adaptive immune responses might be active after 2–3 weeks, and head kidney leucocyte respiratory burst potential (an estimate for innate immune activation) peaks after 7–9 weeks (Barber & Scharsack, 2010; Scharsack et al., 2007). Following those findings, we exposed *S. solidus* infected and sham‐exposed control fish to a defined number of *Diplostomum pseudospathaceum* cercariae 3, 6 and 9 weeks post *S. solidus* infection. The susceptibility to *D. pseudospathaceum* was used as an indicator for the potential systemic modulatory effect of *S. solidus* and interparasitic interactions (Benesh & Kalbe, 2016). *S. solidus’* effect on stickleback immune gene expression was studied in *S. solidus* infected and coinfected hosts (Figure 1).

## 2 MATERIALS AND METHODS

### 2.1 Experimental design

We performed a fully reciprocal coinfection experiment using two pairs of hosts (HR and LR) and *S. solidus* parasites (HG and LG) with contrasting resistance and growth. The infection success of another parasite species, the eye fluke *D. pseudospathaceum*, and stickleback immune gene expression levels were used as quantitative proxies for *S. solidus* immune modulation. We chose three distinct time points after *S. solidus* infection (week 3, week 6, and week 9) to describe the temporal component of the interaction (Figure 1).

### 2.2 Study system

We used naïve laboratory‐bred first generation progeny of three breeding pairs of each of the two stickleback populations (Table 1). The fish were kept in the institute's aquaria facilities at 18°C, with 16 hr of light per day, and fed a diet of frozen chironomids, copepods and daphnids three times a week. We chose two populations of cestodes (Table 1). *S. solidus* from lake Skogseidvatnet grow consistently faster than *S. solidus* from Neustädter Binnenwasser (Benesh & Kalbe, 2016; Kalbe et al., 2016; Ritter et al., 2017), thus justifying the conceptual names for the two types: HG (high growth) and LG (low growth) *S. solidus*. Two *S. solidus* sibships were used per population. A parasite sibship refers to offspring from one *S. solidus* pair that was bred in vitro (Wedekind et al., 1998; modified after Smyth, 1946). All breeding pairs were weight matched to maximize outcrossing rates (Lüscher & Milinski, 2003). *S. solidus* eggs were stored at 4°C in the dark; hatching was initiated following Dubinina (1980). *Macrocyclops albidus* copepods from laboratory cultures were exposed to single coracidia as the first intermediate host (van der Veen & Kurtz, 2002). The copepods were kept at 18°C with 16 hr of light per day, and microscopically checked for *S. solidus* infection one week after exposure. Singly infected copepods were used for stickleback exposure 16 days post exposure.

Susceptibility to the eye fluke *Diplostomum pseudospathaceum* was used as an ecologically relevant proxy for *S. solidus* immune modulation. We established a pool of *D. pseudospathaceum* shedding snails (intermediate hosts) in the laboratory. The snail species *Limnea stagnalis* exclusively hosts *D. pseudospathaceum* in our sampling area (Faltýnková, Našincová, & Kablásková, 2007). *L. stagnalis* were collected in shallow water at different sampling sites of two water bodies connected to the Plöner See lake district (Table 1; Appendix S1) in September and October 2015. All snails were screened for parasites in the laboratory on the day of sampling and trematodes were identified according to Faltýnková et al. (2007). Exclusively *D. pseudospathaceum* positive snails shedding no cercariae of other species were transferred to 16 L tanks in groups of five and fed ad libitum with green lettuce.

### 2.3 Infection experiment and fish dissection

Fish were individually isolated in 2 L tanks and starved for 24 hr before exposure to single *S. solidus* infected copepods. Control fish were exposed to uninfected copepods. We transferred the fish to treatment (fish family × worm sibship combination) specific 16 L tanks after 48 hr, in order to give enough time for copepod ingestion. The water of the single tanks was filtered to quantify uningested copepods. Each 16 L tank housed 18 individuals at the beginning of the experiment. To avoid any density‐dependent influence on growth (Backiel & Lecren, 1978), fish numbers were maintained by replacing fish that died before exposure to *D. pseudospathaceum* by spine‐clipped naïve individuals from the same stickleback families. Three, six and nine weeks after exposure to *S. solidus,* four fish from every treatment were individually exposed to 100 *D. pseudospathaceum* cercariae. The sticklebacks were isolated in 2 L tanks and starved for 24 hr. *D. pseudospathaceum* cercariae came from a pool of at least 10 snails (Kalbe & Kurtz, 2006; Appendix S1) to overcome *D. pseudospathaceum* genotype‐specific effects. Fish were euthanized 2 days post *D. pseudospathaceum* exposure by an incision to the brain and weighed to the nearest 0.1 mg. The standard length (without fin) was measured to the nearest mm. Head kidneys, liver and spleen were weighed to the nearest 0.1 mg; head kidneys were immediately transferred to RNAlater (Sigma‐Aldrich) and stored at room temperature for 24 hr before freezing at −20°C. The sex was determined for each fish, and body cavities were visually inspected for *S. solidus* infection. If present, plerocercoids were weighed and a parasite index (PI) was calculated as 100× cestode weight/fish weight (Arme & Owen, 1967). Host condition was estimated via the condition factor (CF; 100× fish weight/fish length*^b^* with HR‐ and LR‐population specific exponents *b*; Frischknecht, 1993) and the hepatosomatic index (HSI; Chellappa, Huntingford, Strang, & Thomson, 1995). The splenosomatic index (SSI) and a head kidney index (HKI) were calculated as 100× organ weight/fish weight (Bolger & Connolly, 1989; Kurtz et al., 2004) to estimate immunological activation. *D. pseudospathaceum* infection rates were determined by microscopically counting metacercariae completely within the eye lenses in fish‐isotonic NaCl‐solution.

### 2.4 RNA extraction and cDNA synthesis

Head kidney RNA was extracted with a NucleoSpin 96 kit according to the manufacturer's protocol (Macherey‐Nagel), including on column DNA digestion. Samples were homogenized in lysis buffer with 1% β‐Mercaptoethanol using a Tissue Lyser II (Qiagen) for 2 × 3 min at 30 Hz. RNA purity was verified by ensuring all A260/A280 ratios were >1.95 using a NanoDrop 1000 (Thermo Scientific) spectrophotometer. Reverse transcription reactions to cDNA were performed using the Qiagen Omniscript RT kit, following the manufacturer's protocol (Appendix S2). The samples were adjusted to 1,000 ng RNA per reaction. Five samples with concentrations between 500 and 1,000 ng were used in the highest possible concentration and showed comparable results to the remaining data set. The cDNA was stored at −20°C until use for quantitative real‐time PCR (qPCR).

### 2.5 qPCR primer selection and establishment

We chose 32 key targets that had either been published before (Brunner et al., 2017; Hibbeler, Scharsack, & Becker, 2008; Robertson et al., 2015; Stutz et al., 2015) or were designed for this study. We designed intron‐spanning primers for *p22phox, mst1ra* and *marco* using Primer 3 (version 4.0.0, http://primer3.ut.ee). All primers were tested on gDNA and cDNA pools of both stickleback populations on a Light cycler II (ABI) with three technical replicates and a negative control using an annealing temperature of 60°C to ensure protocol compatibility. Amplicon specificity was confirmed by melt curve analysis and gel electrophoresis on a 1.5% agarose gel stained with SybrSafe. Exclusively primers with one unambiguous product and negative gDNA amplification or gDNA product of distinct melting temperature were selected for use. PCR products of all primers were sequenced (Appendix S3) and confirmed by querying the ENSEMBL stickleback reference genome using blastn (Aken et al., 2016; Altschul et al., 1997; ENSEMBL version 86).

Five targets were excluded during establishment (Appendix S4, Table S2). We used four reference genes (*b2m*, *ef1a*, *rpl13a* and *ubc*) (Hibbeler et al., 2008) and 23 immune genes categorized by their functionality in the stickleback's immune system: innate immunity (*cd97*, *csf3r*, *il‐1β*, *marco*, *mif1*, *mst1ra*, *nkef‐β*, *p22^phox^*, *saal1*, *sla1*, *tnfr1)*, adaptive immunity (*stat4*, *cd83*, *igm*, *stat6*, *foxp3b*, *il‐16*, *tgf‐β*, *mhcII, tcr‐β),* and complement system (*c7*, *c9*, *cfb*) (Appendix S5 and Table S3). We further defined gene sets characteristic for a Th1 response (*stat4*, *tnfr1*), Th2 response (*stat6*, *cd83*, *igm*) and Treg response (*il16*, *foxp3*, *tgf‐β*).

### 2.6 Gene expression data acquisition

Relative gene expression was measured with Fluidigm 96.96 Dynamic Array integrated fluidic circuits (IFCs) and Biomark HD system using EvaGreen as DNA binding dye. The initial primer concentration was 100 µM (Appendices S6 and S7). In total, 210 samples were analyzed on four different IFCs. Samples of all treatment groups and time points were randomly distributed across IFCs. Each IFC included two inter‐run calibrators (IRCs) and a gDNA contamination control. Amplification efficiencies were calculated from serial dilutions of HR and LR cDNA pools in a dilution range from 1:10 to 1:10^4^. Primer efficiencies were in the range of 95%–112%, with an *R* ^2^ average value of 0.96 *SE* ± 0.013 (Table S3). Assessment of data quality, reference gene stability, inter‐run calibration and calculation of relative expression values was completed using *qBase+ *3.0 (Biogazelle) (Hellemans, Mortier, De Paepe, Speleman, & Vandesompele, 2007). We set the negative cutoff to the technical sensitivity limit at cycle 28 and allowed a variation of 0.5 cycles for maximum triplicate variability. Expression stability of reference targets was inferred from geNorm M and Coefficient of Variation (CV) values (Hellemans et al., 2007; Vandesompele et al., 2002). The most stably expressed reference genes *rpl13* and *ubc* (*M* = 0.139, CV = 0.049) were used for normalization. Relative expression values were calculated using the ΔΔ*C*_t_ method (Pfaffl, 2001) and exported as log10 transformed CNRQ (calibrated normalized relative quantities). We excluded unreliable data from eight samples. Two missing values for gene *cfb* were replaced by the average *cfb* expression. Accordingly, gene expression analyses were based on 202 infected and control sticklebacks (Appendix S13, Table S11).

### 2.7 Data analyses

Host condition and immunological parameters from 501 sticklebacks were analysed (Appendix S8, Table S4). All statistical analyses were performed in r (version 3.2.0, R Core Team, 2015). We distinguished between time points (T: week 3, week 6, week 9) and host types (H: HR, LR), and defined the following treatment groups (P) for the main analyses: (a) sham‐exposed controls, (b) fish infected with LG *S. solidus*, and (c) fish infected with HG *S. solidus*. We further distinguished between (d) fish infected with *D. pseudospathaceum*, (e) fish coinfected with LG *S. solidus* and *D. pseudospathaceum*, and (f) fish coinfected with HG *S. solidus* and *D. pseudospathaceum*, to analyse host parameters, i.e. condition (CF and HSI) and immunological parameters (SSI and HKI) as well as immune gene expression profiles. Linear mixed effect models (LMMs) and generalized linear mixed effect models (GLMMs) were fit using functions lme() from *nlme* (Pinheiro, Bates, DebRoy, & Sarkar, 2015) and lmer() and glmer() from *lme4* (Bates, Maechler, Bolker, & Walker, 2015). Best fitting models were selected with likelihood ratio tests and the Akaike information criterion (AIC) (Akaike, 1973). *R* ^2^ values of mixed effects models (Johnson, 2014; Nakagawa & Schielzeth, 2013) were calculated with the function sem.model.fits() from *piecewiseSEM* (Lefcheck, 2016). Significantly different groups were identified with glht() post hoc tests from the *multcomp* package (Hothorn, Bretz, & Westfall, 2008) with user defined contrasts according to the respective hypothesis. Apart from that, *p*‐values were obtained with ANOVA() from *car* (Fox & Weisberg, 2011) using Type III Wald chi‐square tests or ANOVA() from *stats* (R Core Team, 2015) computing Type III sum of squares for fixed effects of LMMs. We accounted for multiple testing by using the false discovery rate (FDR, Benjamini & Hochberg, 1995).

Infection rates were compared using GLMMs with binomial error structure and logit link function. *S. solidus* infection rates were analysed with regard to the number of ingested copepods, and included the origin of the fish, the origin of *S. solidus* and their interaction as a fixed structure. Fish origin, *S. solidus* origin, time, and all interactions were tested as fixed effects to analyse *D. pseudospathaceum* infection rates. We additionally tested effects of fish sex, *S. solidus* sibship and fish family, and ultimately incorporated fish family as a random term in the models. To test whether the growth of the worm per se affected *D. pseudospathaceum* infection rates, we used data from *S. solidus* infected fish from each week and added the weight of the worm as a covariate in the statistical models (Benesh & Kalbe, 2016). We included the interaction between worm weight and *S. solidus* origin in the model fit in order to test if the relationship between *S. solidus* growth and susceptibility to *D. pseudospathaceum* was population‐specific. *Schistocephalus* exposed but uninfected fish were excluded from further analyses, because it is not possible to determine the time point and stage of the infection process in which fish resisted infection. LMMs to study *S. solidus* growth, host condition and immunological parameters were fit with fish family as a random term, and heteroscedasticity was accounted for by defining the respective factorial variables as varIdent variance structure. We used parasite indices, the relative weight of the parasite in an infected fish (Arme & Owen, 1967) of all *S. solidus* infected fish (*n* = 140) to study parasite growth over time. The model included the origins of host and parasite, as well as sampling time, and all interactions as fixed effects. Host condition and immunological parameters were analysed with GLMMs using host origin, treatment group (defined above), and sampling time, as well as all interactions as fixed effects.

Stickleback immune gene expression was evaluated by non‐parametric permutational multivariate analyses of variance (PERMANOVA [Anderson, 2001]) on log10 transformed CNRQ values. We first tested if the expression of all 23 immune genes differed between groups within contrasts and, if significant, ran PERMANOVAs according to functional groups (*innate, adaptive, complement; Th1, Th2, Treg*). The analyses were based on Euclidean distances (D'Haeseleer, 2005) using function adonis() from the *vegan* package (Oksanen et al., 2015). The main effects were host type (H), time (T), and depending on the comparison of interest, either treatment group or *S. solidus* type (P). The weight of the fish was included as a covariate to account for size related effects. Each test was based on 10,000 permutations. Permutations were constrained within fish family. Post hoc pairwise comparisons were calculated between contrasts of interest within time points. Experimental treatment effects on single genes of differentially expressed functional groups were tested with LMMs using treatment and fish origin as fixed structure and fish family as random term. Again, we accounted for heteroscedasticity whenever needed and all tests were FDR‐corrected (Benjamini & Hochberg, 1995). Data were plotted with *ggplot2* (Wickham, 2009) and PLYR (Wickham, 2011) using colour schemes from *RColorBrewer* (Neuwirth, 2014). Gene expression was visualized with function aheatmap() from *NMF* (Gaujoux & Seoighe, 2010).

## 3 RESULTS

### 3.1 *S. solidus* growth and effect on stickleback physiology and susceptibility

**Figure 2:**
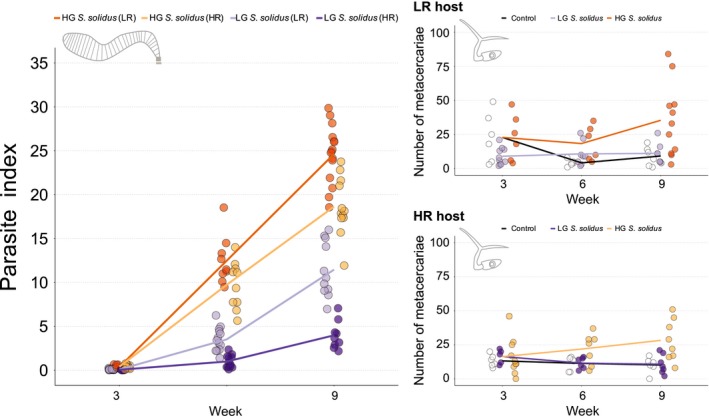
Effect of *S. solidus* growth on susceptibility to *D. pseudospathaceum*. Sticklebacks with either high resistance (HR) or low resistance (LR) were experimentally infected with single *S. solidus* larvae. Parasite indices (parasite weight corrected for host weight) and susceptibility to the eye fluke *Diplostomum pseudospathaceum* (number of metacercariae in the eye lenses 1 day after exposure to 100 cercariae) were determined in week 3, 6, and 9 post *S. solidus* infection. Colour coding follows 1 [Colour figure can be viewed at http://wileyonlinelibrary.com]

*Schistocephalus solidus* infection rates did not differ significantly between host or parasite populations (Appendix S8). The growth of the cestode was significantly affected by *S. solidus* type (Figure 2; Appendix S9, Table S6): high growth (HG) *S. solidus* grew consistently faster than low growth (LG) *S. solidus*. The number of *D. pseudospathaceum* in the eye lenses of sham‐exposed and *S. solidus* infected sticklebacks differed according to a three‐way interaction between time and host and parasite type (Χ^2^_4_ = 24.8413; *p* < 0.0001). Overall, the differences between host populations were not significant (Table S8) and susceptibility to *D. pseudospathaceum* increased over time (Table S9) if sticklebacks were infected with HG *S. solidus*, but not if they were infected with LG *S. solidus* (Figure 2; Table S10). Post hoc comparisons of the effects of parasite type over time and with regard to host type showed that three weeks after *S. solidus* infection, LR hosts had more *D. pseudospathaceum* metacercariae in their eyes if infected with HG *S. solidus* or sham‐exposed, than those infected with LG *S. solidus*; in week 6, *D. pseudospathaceum* numbers in LR fish were highest if hosts were infected with HG *S. solidus* and lowest in controls; in HR hosts, *D. pseudospathaceum* infection rates were significantly higher in HG infected hosts than in controls; 9 weeks after *S. solidus* infection, the number of *D. pseudospathaceum* metacercariae was significantly increased if sticklebacks were infected with HG *S. solidus* (Table S10). We tested if this result was weight‐ rather than population‐specific by fitting GLMMs with *S. solidus* weight as covariate (Appendix S11). At each time point, the number of *D. pseudospathaceum* was not correlated to *S. solidus* weight, and the origin of *S. solidus* remained a significant predictor in week 3 (P effect: Χ^2^_1_ = 6.65, *p* = 0.0099), week 9 (P effect: Χ^2^_1_ = 53.27, *p* < 0.0001), and in LR hosts in week 6 (P effect: Χ^2^_1_ = 4.22, *p* = 0.0401).

Analyses of host condition and immunological parameters are presented in the Supplementary Information (Appendix S12). Briefly, the condition was higher in HR sticklebacks, regardless of the treatment.

### 3.2 Gene expression profiles

**Figure 3:**
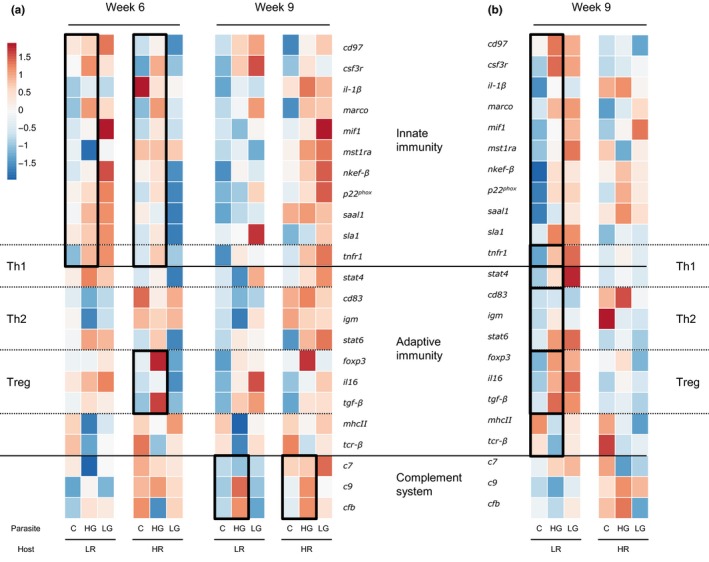
Effects of infection on immune gene expression in sticklebacks over time. Sticklebacks with low resistance (LR) or high resistance (HR) against *S. solidus* were infected with low growth (LG) or high growth (HG) *S. solidus*; controls (C) were sham‐exposed. Heatmaps are based on Euclidean distances of average values of log10‐transformed calibrated normalized relative quantities (CNRQ). Rows are centred and scaled to row *z*‐scores across both host types within weeks. Significantly different groups are highlighted by black outlines. (a) Expression responses in *S. solidus* infected fish after 6 and 9 weeks. (b) Expression responses in *S. solidus* – *D. pseudospathaceum* coinfected fish [Colour figure can be viewed at http://wileyonlinelibrary.com]

Expression profiles of 23 stickleback immune genes were used to characterize the molecular pathways of the host's immune response to *S. solidus* infection over time. We additionally tested for the effects of *D. pseudospathaceum* infection and *D. pseudospathaceum* infection intensity. Multivariate analyses of variance (PERMANOVAs; Anderson, 2001; Brunner et al., 2017) revealed significantly different gene expression profiles of treatment groups over time (Figure 3; Appendix S13). Three weeks after infection, the profiles did not yet differ significantly between *S. solidus* infected and control fish (Table S12). After 6 weeks, HG *S. solidus* infected fish upregulated genes of innate immunity (P effect; PERMANOVA*_innate_*: *F*_1,17_ = 4.9997, *p* = 0.0023), whereas expression profiles of LG‐infected fish did not differ significantly from controls. T regulatory genes were up‐regulated in HG infected HR hosts relative to controls (P effect; PERMANOVA*_Treg_*: *F*_1_,_8_ = 20.14, *p* = 0.0105) (Figure 3a; Table S12). In week 9, genes of complement components were significantly upregulated in HG infected hosts (P effect; PERMANOVA*_complement_*: *F*_1,17_ = 9.899, *p* = 0.0082) (Figure 3a; Table S12). FDR correction of quantitative changes in mRNA levels of single genes indicated significant differential expression of *tgf‐β* in week 6 and *cfb* in week 9 (Tables S13–S15). Multivariate gene expression did not differ significantly between controls and *D. pseudospathaceum* infected fish (Table S16). The profiles differed significantly between controls and LR hosts that were coinfected with *D. pseudospathaceum* and HG *S. solidus*: genes of innate immunity (coinfection effect; PERMANOVA*_innate_*: *F*_1,14_ = 5.43, *p* = 0.0195), adaptive immunity (coinfection effect; PERMANOVA*_adaptive_*: *F*_1,14_ = 5.2, *p* = 0.0122), Th1 (coinfection effect; PERMANOVA*_Th1_*: *F*_1,14_ = 4.8, *p* = 0.0232), Th2 (coinfection effect; PERMANOVA*_Th2_*: *F*_1,14_ = 4.96, *p* = 0.0226) and T regulatory components (coinfection effect; PERMANOVA*_Treg_*: *F*_1,14_ = 11.68, *p* = 0.0074) were upregulated 9 weeks after *S. solidus* infection (Table S17). Primarily, *il‐1β*, *foxp3*, *tgf‐β*, and *il‐16* were higher expressed than in controls (Figure 3b; Table S18). Multivariate gene expression did not differ between coinfected HR fish and the respective controls.

## 4 DISCUSSION

Using controlled experimental helminth infections of three‐spined sticklebacks, we found that proinflammatory, complement and T regulatory pathways are upregulated in chronic infections with a high growth (HG) *Schistocephalus solidus* type after the cestode reached its reproductive weight. Infection rates of another helminth species, the eye fluke *Diplostomum pseudospathaceum* were time‐ and *S. solidus* type‐dependent.

### 4.1 *S. solidus* growth and immune modulation is host and parasite type specific

In a community context, host immunity and parasite virulence are shaped by co‐occurring species such as predators, prey, pathogens and parasites (Schulenburg et al., 2009). We chose hosts and parasites from contrasting environments, where differences in parasite prevalence and diversity potentially selected for host and parasite types with different resistance and virulence (Feulner et al., 2015; Huang et al., 2016; Kalbe et al., 2016). Consistent with previous data (Kalbe et al., 2016), high resistance (HR) host types suppressed parasite growth more than low resistance (LR) host types and high growth (HG) *S. solidus* grew faster than low growth (LG) *S. solidus* in both host types.

Target immune genes were not significantly differentially expressed after 3 weeks, when HG and LG *S. solidus* were small (<3 mg) in both host types. In line with our expectations, LG *S. solidus* were the smallest in every combination and infection rates of *D. pseudospathaceum* were not affected (Figure 2; Appendix S9, Table S5); gene expression profiles of LG‐infected sticklebacks did not differ from controls over the course of the experiment (Figure 3). HG infected sticklebacks increased innate immune responses significantly in week 6, when HG *S. solidus* had reached an average weight of 87 mg in LR hosts and 61 mg in HR hosts (Figure S1; Appendix S9). The proposed minimal weight for sexual reproduction in the final host is 50 mg, and modulatory effects of *S. solidus* are expected above this threshold (Hammerschmidt & Kurtz, 2009; Scharsack et al., 2007; Tierney & Crompton, 1992). HR hosts simultaneously upregulated expression of Treg associated genes, while this regulatory response was absent in LR hosts (Figure 3). We conclude that HG *S. solidus* evolved fast growth in the context of efficient immune modulatory mechanisms in HR hosts, and that HR hosts evolved a well orchestrated immune response to infection.

Later stages of chronic helminth infections are suspected to be accompanied by an activation of the complement system (Haase et al., 2016). Here we found that genes of complement components, especially *cfb*, were only upregulated in HG *S. solidus* infections (Figure 3a), which indicates that the involvement of complement components is *S. solidus* type specific. Helminth genotype‐dependent complement activation was previously proposed for *D. pseudospathaceum* (Haase et al., 2014; Rauch, Kalbe, & Reusch, 2008). It is also tempting to speculate that the ability of the parasite to change its surface composition could involve complement components and leads to evolutionary relevant variation in infectivity and virulence (Hammerschmidt & Kurtz, 2005).

### 4.2 The role of a T regulatory response in HR hosts

A T regulatory response may be beneficial for both host and parasite at late stages of infection as it facilitates survival of the parasite within the stickleback by preventing pathological inflammatory responses (Liu et al., 2010). We monitored expression levels of the Treg related genes *foxp3*, *tgf‐β* and *il‐16* in all treatments over time. FoxP3 (Forkhead Box P3) is a characteristic transcription factor of regulatory T cells; TGF‐β (Transforming growth factor ß) is linked to development of Treg and Th17 cells (Robertson et al., 2015; Weaver, Harrington, Mangan, Gavrieli, & Murphy, 2006). TGF‐β is often classified as a proinflammatory agent despite having regulatory functions (Fischer, Koppang, & Nakanishi, 2013; Liu et al., 2010; Zhu, Nie, Zhu, Xiang, & Shao, 2013). RNA levels of *foxp3* and *tgf‐β* were increased in HR stickleback after 6 weeks. Thus, HG *S. solidus* infected HR hosts upregulated Tregs when the HG parasite initially triggered innate immunity. We conclude that HR hosts, coming from a population with high prevalence of fast growing *S. solidus*, evolved effective resistance and simultaneous upregulation of proinflammatory innate immune genes and T regulatory components, which diminishes negative effects of the cestode or unspecific side effects such as immunopathology. This result is in line with the good condition of HR hosts and in agreement with the recent emphasis on T regulatory functions in helminth infections (Appendix S12; Maizels, 2005; Maizels & McSorley, 2016; Maizels & Yazdanbakhsh, 2003; Nutman, 2015).

### 4.3 Immune gene expression profiles in LR hosts

In stark contrast to the well orchestrated immune response in HG‐infected HR hosts, LR hosts did not upregulate expression of Treg genes upon infection with HG *S. solidus*. Their gene expression response was inefficient: HG and LG *S. solidus* grew faster and condition was lower in LR than in HR hosts. HG *S. solidus*–*D. pseudospathaceum* coinfected LR sticklebacks showed simultaneous significant upregulation of Th1 and Th2 effectors, innate immunity, adaptive immunity and Tregs in week 9. Especially expression levels of *il‐1β*, *foxp3*, *tgf‐β* and *il‐16* were significantly higher than in controls. IL‐16 (Interleukin 16) is a chemoattractant for monocytes and eosinophils, inducing Th1 cell migration and supposedly contributes to Treg cell expansion, for example through the induction of FoxP3 (McFadden et al., 2007; Murphy & Weaver, 2017). Thus, in low resistant LR hosts, two pleiotropic cytokines were highly expressed in combination with proinflammatory molecules during chronic helminth infection. This points towards an ineffective and escalating immune response. We conclude that LR hosts, coming from a population with low *S. solidus* prevalence, cannot mount a concerted and effective immune response when infected with a (HG) *S. solidus* type that evolved fast growth along with strong immune modulation strategies.

### 4.4 *S. solidus* type‐dependent interaction with *D. pseudospathaceum*

Immune gene expression profiles did not differ significantly between *D. pseudospathaceum* infected and control fish, suggesting an effective immune evasion strategy of *D. pseudospathaceum*. The eye fluke migrates to the immune privileged eye lens within 24 hr, thus evades adaptive immunity, and interacts with innate immunity only within this relatively short timeframe (Chappell et al., 1994; Scharsack & Kalbe, 2014). *D. pseudospathaceum* infection rates are therefore determined by the level of immune activation at the moment of infection. Interestingly, *D. pseudospathaceum* infection rates increased over time if hosts were coinfected with HG *S. solidus*. Thus, the *S. solidus* type affects *D. pseudospathaceum* infection success, which could directly or indirectly be mediated through effects on host metabolism or immunity. We expect such effects to be influenced by additional naturally coinfecting parasite species with antagonistic or beneficial effects on the interaction with the host (Benesh & Kalbe, 2016; Telfer et al., 2010). Future laboratory and field experiments (such as those from Benesh & Kalbe, 2016) should thus incorporate additional parasite species in order to study situations closer to the natural setting.

*Diplostomum pseudospathaceum* infection rates were not affected by host immune gene expression if fish had only been infected with this species. Immune gene expression profiles did not differ significantly between host types or between coinfected and control fish until week 9 when HG‐infected LR stickleback simultaneously upregulated genes of most functional groups (Figure 3b).

We cannot conclude whether increased *D. pseudospathaceum* infection rates in HG coinfected hosts were the result of a stress response, cooperation, opportunistic exploitation, or correlation between resistance mechanisms against the two helminth species (Benesh & Kalbe, 2016; Betts et al., 2016). Notably, infection with *D. pseudospathaceum* impairs the vision of infected fish and can cause pathological effects such as increased cataract formation (Karvonen et al., 2004; Meakins & Walkey, 1975). These effects could promote transmission to the final host (fish‐eating birds) of both parasite species through reduction or interference with predator avoidance (Seppälä et al., 2004). *D. pseudospathaceum* infection rates increased after *S. solidus* size was above the expected minimal weight (50 mg) for sexual reproduction (Figure S1; Hammerschmidt & Kurtz, 2009; Tierney & Crompton, 1992). Since fitness of both parasite species relies on transmission to the final host, our data point towards an interaction between *S. solidus* and *D. pseudospathaceum*.

## 5 CONCLUSION

Helminth immune modulation is generally expected to change over the time course of infection but immunological heterogeneity between host populations is often neglected (Benesh & Kalbe, 2016; Maizels & Yazdanbakhsh, 2003; Sitjà‐Bobadilla, 2008). We addressed this knowledge gap by using different naturally co‐occurring helminth species (*S. solidus* and *D. pseudospathaceum*) and types (high growth, HG, and low growth, LG, *S. solidus*) to analyze the immune status of host types from different ecologies and coevolutionary backgrounds with *S. solidus* (high resistance, HR, and low resistance, LR, sticklebacks) over the course of infection. Our results are consistent with the assumption that a well‐orchestrated host response mediates high resistance, namely inhibition of parasite growth (Lohman et al., 2017), and includes mechanisms that protect from immunopathological side effects. We demonstrated that expression profiles can differ between host and parasite types and that coinfection probability of another parasite species increased when the high growth *S. solidus* type reached the proposed minimal weight for sexual reproduction in the final host. Understanding the premises and mechanisms of host‐helminth interactions will advance our knowledge about coevolutionary implications, with potential significance for treatment and prevention strategies in human health and other systems.

## AUTHOR CONTRIBUTION

A.P., M.R., M.K. designed the research and experimental approach. M.K. conceived the study and established and maintained laboratory cultures of hosts and parasites. A.P. and M.R. collected parasite specimens, performed the experiment, analyzed the data and wrote the manuscript.

## Data Availability

All data generated and analyzed in this study is accessible on EDMOND (https://dx.doi.org/10.17617/3.25).
