# Supplementary material for: The right response at the right time: Exploring helminth immune modulation in sticklebacks by experimental coinfection
Source: Mol Ecol. 2019 May 17;28(10):2668–80. doi: 10.1111/mec.15106 (PMC6852435; doi:10.1111/mec.15106)
Supplement: Supplementary file 1 [file MEC-28-2668-s001.pdf]

MOLECULAR ECOLOGY

Supplemental Information for:

The right response at the right time: Exploring helminth immune modulation in sticklebacks by experimental co-infection

Agnes Piecyk, Marc Ritter, Martin Kalbe

Table of Contents:

SI.1 Information on *Diplostomum pseudospathaceum* sampling and use 2
SI.2 Further information on reverse transcription 2
SI.3 Further information on direct sequencing 2
SI.4 Further information on gene expression analyses 2
SI.5 Gene expression targets, gene references and primer sequences 3
SI.6 Pre-amplification of target cDNA for Fluidigm 96.96 Dynamic Array run 6
SI.7 Fluidigm 96.96 Dynamic Array run using pre-amplified cDNA......................................7
SI.8 Further information on sample sizes and *S. solidus* infection rates 7
SI.9 Further information on *S. solidus* growth and parasite index 8
SI.10 Further information on *D. pseudospathaceum* infection rates 9
SI.11 The effect of *S. solidus* weight on *D. pseudospathaceum* infection rates................11
SI.12 Further information on host condition and immunological parameters 11
SI.13 Detailed results of gene expression analyses 11
References 17

SI.1 Information on *Diplostomum pseudospathaceum* sampling and use

Limnea stagnalis were sampled by hand or using a small dip net. In the laboratory, each snail was subsequently rinsed with filtered lake water and individually placed in a plastic cup (Bioware 200ml, Huhtamaki) with filtered lake water. After two hours of direct light exposure, snails were screened for trematode infections by inspecting the shed cercariae in the water of the cup. The snails were kept in 16L aquaria at 18°C, with 16 hours of light per day. We used clone mixes from a pool of snail hosts in every infection round in order to overcome strong influences of *D. pseudospathaceum* genotype specificities: following a recovery period of at least two weeks post sampling, *D. pseudospathaceum* positive snails were individually placed in plastic cups with filtered lake water and exposed to direct light for 60 minutes. After verification of infection status and snail viability, 10 snails shedding the largest number of cercariae of the day were transferred to new plastic cups with fresh lake water and exposed to direct light for another 60 minutes. Cercariae from this supernatant were used to create a pool of *D. pseudospathaceum* cercariae of similar age.

SI.2 Further information on reverse transcription

The reverse transcription protocol was modified by using 0.2 µL Qiagen RNAse inhibitor (instead of 1 µL). The manufacturer ensured that 0.2 µL is sufficient due to differences in effective inhibitor concentrations.

SI.3 Further information on direct sequencing

PCR conditions were the same in all sequencing attempts. All PCR products were checked on a gel for the right size and amplification specificity. 5 µL aliquot of the completed PCR reaction were mixed thoroughly. 2 µL of Illustra ExoStar 1 Step were added to the reaction mix and incubated at 37°C for 15 minutes. Incubation at 80°C for 15 minutes inactivated the enzymes. Afterwards, the cycle sequencing was prepared as follows:

2 µL PCR/Product /ExoSAP + 2 µL Seqbuffer+1 µL Primer (forward or reverse of each PCR primer) + 4 µL HPLC H^2O + 1 µL BDT

Program: BDT 3.1

Cycle	Temp	Min
Pre-denaturation	96°C	01:00
Denaturation	96°C	00:10
Annealing	60°C	04:00
	4°C	∞

cleaned up with BigDye XTerminator® Purification Kit from Applied Biosystems
sequenced on 3130 XL Genetic Analyzer from Applied Biosystems

SI.4 Further information on gene expression analyses

Table S2. Excluded primers

Gene	Function	References	comment
tlr2	Toll-like receptor 2; Germline-encoded pattern-recognition receptor	(Zhu et al., 2013; Brunner et al., 2017)	Amplification efficiency of primer product was not within acceptable range
p40phox	Component of NADPH oxidases	(Stutz et al., 2015)	Product sequencing revealed amplification of unspecific target
vegfa1	Stimulates macrophage and monocyte migration	(Brunner et al., 2017)	Unspecific primer products, ambiguous PCR products
ly75	Reduces B-lymphocyte proliferation	(Brunner et al., 2017)	Unspecific primer products, ambiguous PCR products
cmip	Signaling protein in Th2 pathway	(Robertson et al., 2015)	Unspecific primer products, ambiguous PCR products

SI.5 Gene expression targets, gene references and primer sequences

Gene	Function	References	ENSEMBL ID	Forward primer	Reverse primer
**Reference genes**					
b2m	Beta-2-microglobulin	(Hibbeler et al., 2008)	ENSGACT00000025544	GAAGATGTGTTGAATAGAAGCTGG	GAAGATGTGTTGAATAGAAGCTGG
ef1a	Elongation factor 1 α	(Hibbeler et al., 2008)	ENSGACT00000002893	CCACCGTTGCCTTTGTCC	TGGGACTGTTCCAATACCTCC
rpl13a	L13A ribosomal binding protein	(Hibbeler et al., 2008)	ENSGACT00000012319	CACCTTGGTCAACTTGAACAGTG	TCCCTCCGCCCTACGAC
ubc	Ubiquitin	(Hibbeler et al., 2008)	ENSGACT00000010662	AGACGGGCATAGCACTTGC	CAGGACAAGGAAGGCATCC
**Innate**					
cd97	Promotor of granulocyte and neutrophil migration, required for activation of the innate immune response	(Leemans et al., 2004; Rhodes et al., 2009)	ENSGACT00000024871	CTCGTGGCACTCTACGACATGAAG	CAGCCCTATCTTGGTGACCAGTTG
csf3r	Granulocyte colony-stimulating factor 3 receptor; role in differentiation and proliferation of granulocytes	(Tabbara, 1993; Birrer et al., 2012; Maxson et al., 2013; Brunner, 2016)	ENSGACT00000018254	TCGGGATTCGTCCTCTTCTCAG	TGGGTCAAACTTGGCTGCAC
il-1β	Interleukin 1 β; cytokine with function in early response proinflammatory signaling	(Zhu et al., 2013; Brunner et al., 2017)	ENSGACT00000019325	TGACGATGAAGCAGGTGGTCAAC	ACAGCGTCACGATCTCCTCTTC
marco, RON	Macrophage receptor with collagenous structure; mediates macrophage recognition and clearance of pathogens	(Kraal et al., 2000; Kissick et al., 2014)	ENSGACT00000001965	CCCTTTCGACCTTCACTGCC	TGTTTACCCCAACCCCTCCA
mif1	Macrophage migration inhibitory factor; stops random macrophage migration through tissue, proinflammatory mediator of the innate immune system	(Calandra & Roger, 2003; Brunner et al., 2017)	ENSGACT00000023656	ATCAGCGGAGCTCACAACAAGC	TCAGGAGAGATGCTCAGGTGTTTG
mst1ra	Macrophage stimulating 1 receptor a; plays an important role in macrophage regulation	(Wang et al., 2002; Huang et al., 2016)	ENSGACT00000013997	ATGGCCATCGAAAGCTTGCA	TGATGTCGTACGGGTCCACA

nkef-β, peroxi-redoxin 1	Natural killer cell enhancing factor; enhances cytotoxicity of NK cells, also protects against oxidative damage	(Shau et al., 1993; Stutz et al., 2015)	ENSGACT00000021380	ACTTCTCCC ACTTTGCATGG	CAATGCCTTCATCCTCCTTC
p22phox	NADPH oxidase component p22phox; part of the reactive oxygen species production machinery	(Bedard & Krause, 2007; Mayumi et al., 2008)	ENSGACT00000021084	GCCTCGGGACTCATTCTCCT	TGGCCCTCTTGCTTCTTGGA
saal1	Serum amyloid A; acute phase protein during inflammation response, mediates release of TNF-α and IL-1β	(Haarder et al., 2013; Kovacevic et al., 2015; Brunner et al., 2017)	ENSGACT00000007599	TCGCAGTGAGGCCAAAGATGAG	AAATCTGCCACCGTGTCCTTGG
sla1	Src-like-adaptor; necessary for maturation and activation of monocytic and dendritic cells, functions in T-cell signaling and B-cell development and function	(Marton et al., 2015; Brunner et al., 2017)	ENSGACT00000007895	ACAGAGTCGGCTCCTTCATGATAC	TCACAGAGAGCGAATACAGACCTC
tnfr1	Tumor necrosis factor receptor 1; functions in regulation of inflammation, mediates cellular apoptosis and differentiation	(Zhu et al., 2013; Brunner et al., 2017)	ENSGACT00000013502	AACTACTACAGAGCCAAGGGCAAG	ACGGCACTCAGCGGTACAATTC
**Adaptive**					
cd83	Marker for mature dendritic cells, expressed on activated B and T cells, costimulatory to activate naïve and memory T-cells	(Aerts-Toegaert et al., 2007; Stutz et al., 2015)	ENSGACT00000000428	AGGACCCAGCGTATAAATGG	CCCTGGTGATTTTCCTCATC
foxp3; forkhead box N2b	Transcription factor; regulates functions important for the establishment of the T-reg lineage, key mediator of T-cell activation	(Rao & Naqvi, 2011; Robertson et al., 2015; Kasheta et al., 2017)	ENSGACT00000007261	GTTGACCCATGCAATTCCGA	CTGCTGTAGTTGTGGTCCTG
igm	Immunoglobulin heavy constant mu (IgM); antibody molecule, part of the humoral immune response	(S. Hibbeler, unpublished; Rønneseth et al., 2015; Zhu et al., 2013)	ENSGACT00000016907	AAGGCAGGAGAATGAAACCTTGG	CCGAGTGAGCAGACAGGGACTGG
il-16	Interleukin 16; cytokine with function in T-cell migration and expansion, chemoattractant for monocytes and eosinophils	(Wen et al., 2006; Zhu et al., 2013; Brunner et al., 2017)	ENSGACT00000016499	CTGGTCTGGGCTTCAGTATTGC	CTGGGAAACACTCTGTGGACTG

mhcII	Major histocompatibility complex class IIb exon 2; pathogen recognizing protein of the adaptive immune response,	(Lenz et al., 2009)	ENSGACT00000000425	GTCTTTAACTCCACGGAGCTGAAGG	ACTCACCGGACTTAGTCAG
stat4	Signal transducer and activator of transcription 4; required for TH1-cell differentiation, opposes TH2 and TH17 like responses	(Kaplan, 2005; Premachandra et al., 2013; Wang & Secombes, 2013)	ENSGACT00000003538	CTCTCAGTTTCGAGGCTTGCTT	GGCAGTTGGCTCACATTGG
stat6	Signal transducer and activator of transcription 6; required for TH2-cell differentiation, regulates expression of TH2 relevant cytokine IL-4	(Wang & Secombes, 2013; Robertson et al., 2015)	ENSGACT00000011232	CTCAGCCACAGTTCCAACCGTTC	GTCGGATGTTCTGGACCTCGAGT
tcr-β	T-cell receptor β-chain; function in binding of MHC-peptide ligands to initiate adaptive immune response	(Yanagi et al., 1984; Smith-Garvin et al., 2009; Stutz et al., 2015)	ENSGACT00000016457	GAGGGCAAAAACTTCACCTG	TAGGAGAATCTGGCCGTTTG
tgf-β	Transforming growth factor β; cytokine with functions in cell growth, migration, differentiation and proliferation of T and B-cells	(Zhu et al., 2013; Robertson et al., 2015)	ENSGACT00000016968	TCCCGCTTCGTCACCAACCA	ACGTCTGTCTGGCCACATTCAC
**Complement**					
c7	Complement component 7; initializing function in the membrane attack complex of the complement system	(Zhu et al., 2013; Haase et al., 2014; Brunner et al., 2017)	ENSGACT00000009181	TGGCTCAAGCTCAGCACAACAG	AGCGACACGTGTTTGTTTGATCG
c9	Complement component 9; structural part of the membrane attack complex of the complement system,	(Zhu et al., 2013; Haase et al., 2014; Brunner et al., 2017)	ENSGACT00000020968	CCGTGACGAACAAAGACTCAGTTG	TCTGACCGATGTCAGCACCTTG
cfb	Complement factor B; activating complement component of the alternative pathway	(Zhu et al., 2013; Haase et al., 2014; Brunner, 2016)	ENSGACT00000027346	GAGCGTCGCACAATACAGGTTG	TACCACCGGAAGCGCACAAATC

Table S3. Primer efficiencies

Primer ID	Efficiency	E (SE)	R^2
cfb	1.909	0.093	0.803
c7	2.089	0.015	0.995
c9	1.948	0.126	0.742
cd83	2.047	0.033	0.978
csf3r	2.024	0.085	0.885
foxp3	2.065	0.028	0.986
cd97	2.029	0.05	0.957
igm	2.087	0.017	0.994
mhcII	2.011	0.024	0.988
mif1	2.064	0.015	0.995
nkef-β	2.144	0.016	0.996
sla1	2.161	0.017	0.996
stat4	2.243	0.028	0.99
stat6	2.062	0.024	0.989
tcr-β	2.02	0.014	0.996
tgf-β	2.079	0.02	0.994
tnfr1	2.19	0.025	0.99
il-16	2.163	0.018	0.994
il-1β	2.016	0.101	0.852
marco	2.092	0.02	0.993
mst1ra	2.228	0.042	0.976
p22phox	2.006	0.012	0.996
rpl13a	2.048	0.022	0.992
saal1	2.025	0.034	0.974
ubc	2.116	0.014	0.996

SI.6 Pre-amplification of target cDNA for Fluidigm 96.96 Dynamic Array run

- Primer Mix: total 200 µL
- 1 µL of each 100µM primer (fwd and rev) or 2 µL of paired primer mix
- plus 136 µL DNA suspension buffer (10 mM Tris, pH 8.0, 0,1 mM EDTA)

Pre Mix - Prepared in a 1.5 ml tube: total 396 µL (includes overage)

- 264 µL 2X TaqMan PreAmp Master Mix (Applied Biosystems, PN 4391128)
- 52.8 µL Primer Mix
- 79.2 µL H_2O

We pipetted 3.7 µL Pre-Mix in each well of a 96 well plate and added 1.3 µL of cDNA. Negative controls (NTCs) were included by using 1.3 µL of ddH_2O instead of cDNA. The PCR protocol was the following:

<table>
<tr><th>Temp</th><th>Time</th><th>No. cycles</th></tr>
<tr><td>95 °C</td><td>10 min</td><td></td></tr>
<tr><td>95 °C</td><td>15 sec</td><td rowspan="2">14</td></tr>
<tr><td>60 °C</td><td>4 min</td></tr>
<tr><td>4 °C</td><td>∞</td><td></td></tr>
</table>

SI.7 Fluidigm 96.96 Dynamic Array run using pre-amplified cDNA

Pre Mix – prepared in a 1.5 ml tube: total 406.6 µL (for 96 samples, includes overage)
- 369.6 µL SsoFast EvaGreen Supermix with Low ROX (BioRad, PN 172-5211)
- 37 µL 20X DNA Binding Dye Sample Loading Reagent (Fluidigm, PN 100-3738)

The following was pipetted into each well of a 96 well plate
- 3.9 µL Sample Pre Mix
- 3.1 µL sample (preamplified)
- vortexed 20s, spun down 30s

Assay Pre-mix – prepared in a 1.5 ml tube: total 665.3 µL (for 96 reactions, includes overage)
- 369.6 µL 2X Assay loading Reagent (Fluidigm, PN 85000736)
- 295.7 µL low TE buffer

The following was pipetted into each well of a 96 well plate (7 µL per well)
- 6.3 µL Assay Pre-mix
- 0.35 µL from each of the 100µM primers (fwd and rev) or 0.7 µL from the mix

After priming of the chip, Sample Pre Mix and Assay pre Mix were loaded according to the manufacturer's instructions and the chip was run under cycler protocol: "GE Fast 96x96 PCR+Melt v2".

Temp	Time	No. Cycles
70 °C	40 min	
60 °C	30 sec	
95 °C	1 min	
96 °C	5 sec	30
60 °C	20 sec	
60 - 95 °C	+ 1°C/3s	

SI.8 Further information on sample sizes and *S. solidus* infection rates

Table S4. Sample sizes

Treatment group	week 3	week 6	week 9
Sham-exposed HR control	6	6	6
Sham-exposed LR control	6	6	4
HR hosts infected with LG *S. solidus* (single and co-infections)	8	12	9
HR hosts exposed to LG *S. solidus* (uninfected)	28	24	22
LR hosts infected with LG *S. solidus* (single and co-infections)	16	13	11
LR hosts exposed to LG *S. solidus* (uninfected)	20	23	24
HR hosts infected with HG *S. solidus* (single and co-infections)	16	10	12
HR hosts exposed to HG *S. solidus* (uninfected)	20	26	19
LR hosts infected with HG *S. solidus* (single and co-infections)	9	9	15
LR hosts exposed to HG *S. solidus* (uninfected)	27	27	19
HR hosts infected with *D. pseudospathaceum*	6	6	6
LR hosts infected with *D. pseudospathaceum*	6	6	6
Fish that died before dissection		12	
Total		501	

We tested effects on *S. solidus* infection rates by using a GLMM with the origin of the host and the origin of the parasite as well as their interaction as fixed effects and fish family as random term. The model did not differ significantly from the Nullmodel (likelihood ratio test: X^2_3 = 4.2365, p = 0.237).

SI.9 Further information on *S. solidus* growth and parasite index

S. solidus growth differed significantly between the two parasite populations. HG parasites grew faster and larger than LG parasites (Type III Wald chisquare tests: H:P:T three-way interaction: X^2_4 = 24.8413, p < 0.0001).

Table S5. Differences in *S. solidus* parasite indices according to host types

Contrast					
T	P:H	Estimate	Std. Error	z value	Pr(>|z|)
3	LG:HR - LR	-0.04425	0.06905	-0.641	0.98779
3	HG:HR - LR	0.03158	0.06729	0.469	0.99773
6	LG:HR - LR	-2.52061	0.77996	-3.232	0.00736
6	HG:HR - LR	-2.74607	0.89502	-3.068	0.01284
9	LG:HR - LR	-7.48326	1.34521	-5.563	< 1e-05
9	HG:HR - LR	-6.00136	1.15927	-5.177	< 1e-05

Table S6. Differences in *S. solidus* parasite indices according to *S. solidus* types

Contrast					
T	P:H	Estimate	Std. Error	z value	Pr(>|z|)
3	HG - LG:LR	0.27701	0.05873	4.717	1.44E-05
3	HG - LG:HR	0.35284	0.06169	5.72	6.40E-08
6	HG - LG:LR	8.96403	0.8441	10.62	< 1e-10
6	HG - LG:HR	8.73858	0.83369	10.482	< 1e-10
9	HG - LG:LR	13.17036	1.18776	11.088	< 1e-10
9	HG - LG:HR	14.65227	1.31937	11.105	< 1e-10

Table S7. Differences in parasite indices over time

Contrast					
T	P:H	Estimate	Std. Error	z value	Pr(>|z|)
6 - 3	LG:LR	3.4222	0.541	6.325	< 1e-04
6 - 3	LG:HR	0.9459	0.5643	1.676	0.593759
6 - 3	HG:LR	12.1092	0.6505	18.614	< 1e-04
6 - 3	HG:HR	9.3316	0.6168	15.129	< 1e-04
9 - 3	LG:LR	11.3698	0.9028	12.594	< 1e-04
9 - 3	LG:HR	3.9308	0.9986	3.936	0.000904
9 - 3	HG:LR	24.2632	0.7739	31.35	< 1e-04
9 - 3	HG:HR	18.2303	0.8645	21.087	< 1e-04
9 - 6	LG:LR	7.9476	1.0514	7.559	< 1e-04
9 - 6	LG:HR	2.985	1.1448	2.607	0.087956
9 - 6	HG:LR	12.1539	1.0089	12.047	< 1e-04
9 - 6	HG:HR	8.8987	1.0606	8.39	< 1e-04

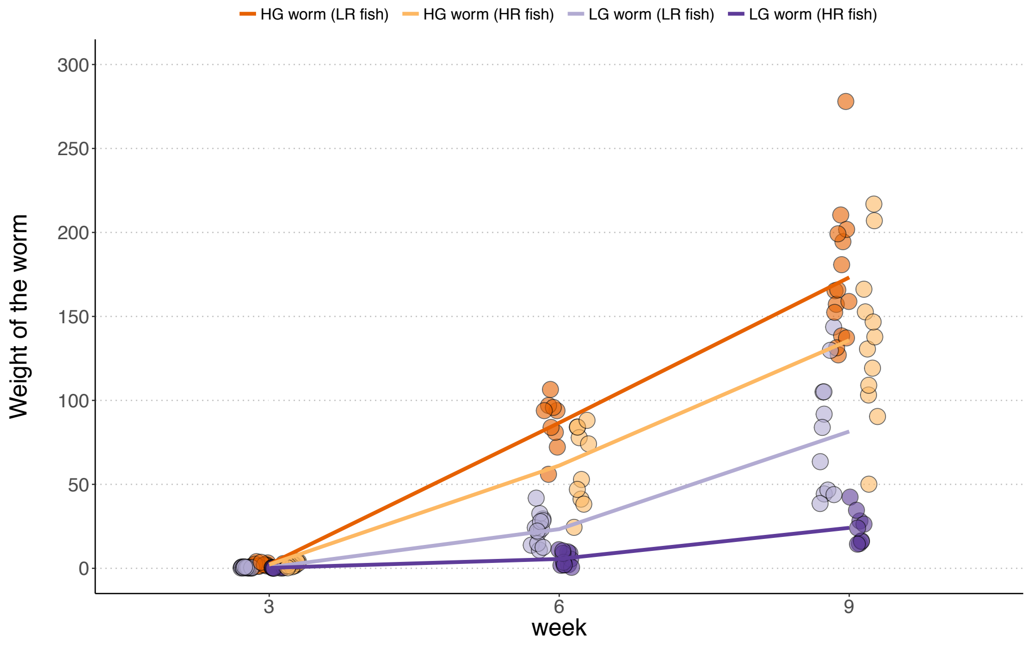

Fig. S1. *Schistocephalus solidus* growth over time. Plerocercoids were weighed 3, 6, or 9 weeks after infection. Each dot represents one *S. solidus* individual; color coding follows Fig. 1. Weights were measured in mg. HG: high growth *S. solidus*; LR: low resistance stickleback; LG: low growth *S. solidus*; HR: high resistance stickleback.

SI.10 Further information on *D. pseudospathaceum* infection rates

Table S8. *D. pseudospathaceum* infection rate differences between host types

	Contrast				
T	P:H	Estimate	Std. Error	z value	Pr(>|z|)
3	CTRL:HR - LR	-0.5708	0.516	-1.106	0.5222
3	LG:HR - LR	0.4501	0.52	0.866	0.696
3	HG:HR - LR	-0.124	0.5099	-0.243	0.9994
6	CTRL:HR - LR	1.2871	0.5485	2.347	0.0541
6	LG:HR - LR	0.6251	0.5226	1.196	0.4638
6	HG:HR - LR	0.422	0.5131	0.822	0.7289
9	CTRL:HR - LR	0.2924	0.5298	0.552	0.9212
9	LG:HR - LR	0.1643	0.5221	0.315	0.9962
9	HG:HR - LR	-0.1888	0.5022	-0.376	0.9883

Table S9. ***D. pseudospathaceum*** **infection rate differences over time**

Contrast					
T	P:H	Estimate	Std. Error	z value	Pr(>|z|)
6 - 3	CTRL:LR	-2.03113	0.23082	-8.799	< 1e-05
6 - 3	CTRL:HR	-0.17322	0.17765	-0.975	0.99532
6 - 3	LG:LR	-0.24223	0.1645	-1.473	0.88485
6 - 3	LG:HR	-0.06721	0.19189	-0.35	1
6 - 3	HG:LR	-0.29627	0.14855	-1.994	0.512
6 - 3	HG:HR	0.24966	0.13211	1.89	0.59702
9 - 3	CTRL:LR	-1.15953	0.17763	-6.528	< 1e-05
9 - 3	CTRL:HR	-0.2964	0.18226	-1.626	0.79653
9 - 3	LG:LR	-0.09894	0.16859	-0.587	0.99997
9 - 3	LG:HR	-0.38468	0.17346	-2.218	0.34321
9 - 3	HG:LR	0.78552	0.12199	6.439	< 1e-05
9 - 3	HG:HR	0.72067	0.12039	5.986	< 1e-05
9 - 6	CTRL:LR	0.8716	0.25233	3.454	0.00949
9 - 6	CTRL:HR	-0.12318	0.18768	-0.656	0.99991
9 - 6	LG:LR	0.14329	0.16962	0.845	0.99879
9 - 6	LG:HR	-0.31746	0.18746	-1.694	0.74999
9 - 6	HG:LR	1.08179	0.12868	8.407	< 1e-05
9 - 6	HG:HR	0.47101	0.12281	3.835	0.0022

Table S10. ***D. pseudospathaceum*** **infection rate differences between** ***S. solidus*** **types**

Contrast					
T	P:H	Estimate	Std. Error	z value	Pr(>|z|)
3	LG - CTRL:LR	-0.98089	0.15285	-6.417	< 1e-05
3	LG - CTRL:HR	0.03994	0.17581	0.227	1
3	HG - CTRL:LR	-0.29524	0.14467	-2.041	0.472444
3	HG - CTRL:HR	0.15155	0.15047	1.007	0.993599
3	HG - LG:LR	0.68564	0.15291	4.484	0.000134
3	HG - LG:HR	0.11161	0.15733	0.709	0.999787
6	LG - CTRL:LR	0.80801	0.23732	3.405	0.011265
6	LG - CTRL:HR	0.14595	0.18638	0.783	0.999416
6	HG - CTRL:LR	1.43961	0.23358	6.163	< 1e-05
6	HG - CTRL:HR	0.57443	0.16127	3.562	0.006328
6	HG - LG:LR	0.63161	0.1596	3.957	0.001328
6	HG - LG:HR	0.42848	0.17063	2.511	0.175896
9	LG - CTRL:LR	0.0797	0.19027	0.419	0.999999
9	LG - CTRL:HR	-0.04833	0.18436	-0.262	1
9	HG - CTRL:LR	1.64981	0.15982	10.323	< 1e-05
9	HG - CTRL:HR	1.16862	0.15769	7.411	< 1e-05
9	HG - LG:LR	1.57011	0.14074	11.156	< 1e-05
9	HG - LG:HR	1.21696	0.14675	8.293	< 1e-05

SI.11 The effect of *S. solidus* weight on *D. pseudospathaceum* infection rates

Using *S. solidus* weight as a covariate in the statistical model did not improve the model fit in week 3, but did so at later time points, namely for data from LR fish in week 6 (likelihood ratio test: $X^2_2 = 10.01$, $p = 0.0067$) and data from both fish origins in week 9 (likelihood ratio test: $X^2_2 = 13.37$, $p = 0.0013$). The model fit for HR data of week 6 was not improved (likelihood ratio test: $X^2_2 = 4.82$, $p = 0.0897$). Due to very large eigenvalues, we z-transformed the weight of the worm in week 6 and week 9.

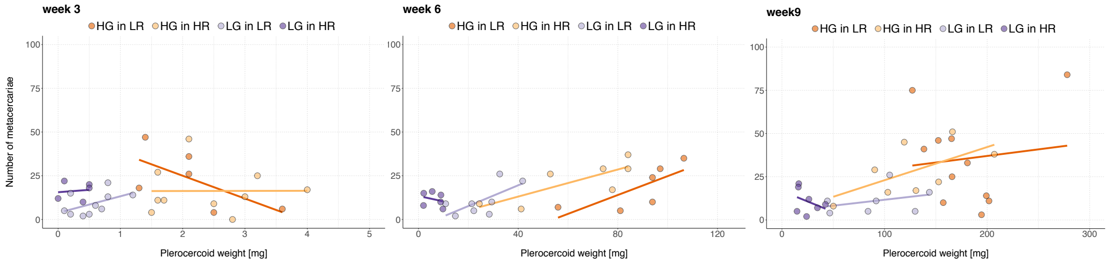

Figure S2. The relationship between *S. solidus* weight and *D. pseudospathaceum* infection rates. *S. solidus* infected sticklebacks were exposed to 100 *D. pseudospathaceum* cercariae at three different time points. Each dot represents one *S. solidus* individual; lines represent linear model fits; color coding follows Fig. 1.

SI.12 Further information on host condition and immunological parameters

The condition factor (CF) differed significantly between host populations ($F_{1,4} = 25.027$, $p = 0.0075$) and according to an interaction between time point and treatment ($F_{10,170} = 2.543$, $p = 0.007$). FDR-corrected post hoc comparisons confirmed significantly higher condition of HR than LR hosts at all time points (LMMs; each $p < 0.0001$). Treatment had no significant influence; the CF increased between week 3 and week 9 in *D. pseudospathaceum* infected HR fish. The hepatosomatic index (HSI) was significantly affected by an interaction between treatment and time point ($F_{10,170} = 4.102$, $p < 0.0001$). LR controls had higher HSIs than HR controls in week 9; the HSI increased between week 3 and 9 in LR controls (LMMs; each $p < 0.001$). In week 9, LG infection was associated with a smaller HSI than in controls in LR fish; infection with *D. pseudospathaceum* correlated with significantly higher HSI in comparison to co-infection with HG *S. solidus* in LR sticklebacks (LMMs; each $p < 0.001$). Splenosomatic indices (SSI) and head kidney indices (HKI) were not affected by experimental factors.

SI.13 Detailed results of gene expression analyses

Table S11. Sample sizes for gene expression analyses

Treatment group	week 3	week 6	week 9
Sham-exposed HR control	6	6	6
Sham-exposed LR control	4	6	4
HR hosts infected with LG *S. solidus*	3	6	2
LR hosts infected with LG *S. solidus*	6	5	4
HR hosts infected with HG *S. solidus*	5	3	4
LR hosts infected with HG *S. solidus*	3	3	4
HR hosts infected with *D. pseudospathaceum*	5	5	6
LR hosts infected with *D. pseudospathaceum*	5	6	5
HR hosts co-infected with LG *S. solidus* & *D. pseudospathaceum*	4	6	7
LR hosts co-infected with LG *S. solidus* & *D. pseudospathaceum*	10	8	7
HR hosts co-infected with HG *S. solidus* & *D. pseudospathaceum*	10	7	8
LR hosts co-infected with HG *S. solidus* & *D. pseudospathaceum*	6	6	11
Total		202	

Table S12. Multivariate statistics (PERMANOVA results) of *S. solidus* infection effect on stickleback immune gene expression

contrast							
T	P:H		Df	SumsOfSqs	F.Model	Pr(<F)	R2
3	LG - CTRL:HR&LR	all genes	1	2.720	1.27410	0.3787	0.06220
3	HG - CTRL:HR&LR	all genes	1	1.813	0.6536	0.5375	0.03490
3	HG - LG:HR&LR	all genes	1	1.180	0.6698	0.6554	0.03258
6	LG - CTRL:HR&LR	all genes	1	0.683	0.3826	0.8597	0.01536
**6**	**HG - CTRL:HR&LR**	all genes	1	6.037	3.08185	0.0081	0.15855
		innate	1	4.7112	4.9997	**0.0023**	0.23481
		adaptive	1	0.9015	3.9718	0.0176	0.19101
		complement	1	0.4241	0.53716	0.5240	0.03191
		Th1	1	0.27172	6.3830	0.0281	0.28856
		Th2	1	0.21162	1.41429	0.2842	0.08102
		Treg (P effect)	1	0.2842	11.4826	0.0003	0.32809
		Treg (H effect)	1	0.09366	4.3531	0.0222	0.12438
		Treg (P:H interaction)	1	0.09119	4.2384	0.0240	0.12110
6	HG - CTRL:LR	*Treg*	1	0.035747	1.11233	0.3463	0.13857
**6**	**HG - CTRL:HR**	***Treg***	1	0.28071	20.1432	**0.0105**	0.72529
6	HG - LG:HR&LR	all genes	1	0.857	0.45844	0.79982	0.02550
9	LG - CTRL:HR&LR	all genes (P effect)	1	1.979	1.4393	0.05411	0.06145
		all genes (H effect)	1	5.580	4.0580	0.01128	0.17326
9	LG - CTRL:LR	all genes	1	2.6294	1.7392	0.07292	0.18035
9	LG - CTRL:HR	all genes	1	1.8375	1.5914	0.2361	0.15425
9	HG - CTRL:HR&LR	all genes	1	16.972	5.8497	0.00340	0.27223
		innate	1	2.9039	2.16177	0.07469	0.11711
		adaptive	1	0.7096	3.4025	0.13248	0.0393
		complement	1	13.358	9.8992	**0.0082**	0.41497
		Th1	1	0.14091	1.90958	0.1356	0.11422
		Th2	1	0.17302	1.9625	0.1893	0.06081
		Treg	1	0.10111	2.99380	0.07879	0.15223
9	HG - LG:HR&LR	all genes	1	11.363	3.4461	0.07465	0.21681

The statistical models were based on log10-transformed calibrated normalized relative quantities (CNRQ values). The weight of the fish was included as covariate to account for size related effects. Non-parametric permutational multivariate analyses of variance (PERMANOVA) were calculated on Euclidean distances and 10,000 permutations that were constrained within fish family. PERMANOVA results were FDR corrected. If significant (marked in bold letters), single genes were analyzed with linear mixed models (LMMs). Statistics for differences between host types or interactions are mentioned whenever significant. T: time point (week 3, 6, or 9); P: parasite type (low growth, LG; high growth, HG); H: host type (low resistance, LR; high resistance, HR); *all genes*: data from all 23 genes; *innate*: 11 genes (*cd97*, *csf3r*, *il-1β*, *marco*, *mif1*, *mst1ra*, *nkef-β*, *p22^{phox}*, *saal1*, *sla1*, *tnfr1*); adaptive: nine genes (*stat4*, *cd83*, *igm*, *stat6*, *foxp3*, *il-16*, *tgf-β*, *mhcII, tcr-β*); *complement*: three genes (*cfb*, *c7*, *c9*); *Th1*: two genes (*stat4*, *tnfr1*), *Th2* covers three genes (*stat6*, *cd83*, *igm*); *Treg* covers three genes (*il-16*, *foxp3*, *tgf-β*).

Table S13. Differential innate immune gene expression between HG-*S. solidus* infected and control (HR and LR) stickleback in week 6

ANOVA results	numDF	denDF	F-value	p-value	pseudo R2
marco	1	9	0.193756	0.6702	0.4923706
mst1ra	1	9	1.88193	0.2033	0.9672328
mif1	1	9	3.305022	0.1024	0.4501657
il-1β	1	9	0.400218	0.5427	0.4503203
tnfr1	1	9	6.234099	0.0340	1
saal1	1	9	6.068530	0.0360	0.5988541
csf3r	1	9	2.0903358	0.1821	0.427818
p22phox	1	9	1.250352	0.2924	0.512474
nkef-β	1	9	0.0634747	0.8067	0.09538045
sla1	1	9	1.2502136	0.2925	0.4952347
cd97	1	9	0.347441	0.5701	0.3215247

The statistical models were based on log10-transformed calibrated normalized relative quantities (CNRQ values). The weight of the fish was included as covariate to account for size related effects. Data from genes from significantly differentially expressed functional gene groups was analyzed with linear mixed models (LMMs; function lme() from *nlme*) and analyses of variance (ANOVAs). Conditional pseudo R^2 values (Nakagawa & Schielzeth, 2013; Johnson, 2014) were calculated with sem.model.fits() from *piecewiseSEM* (Lefcheck, 2015). No gene was significantly differentially expressed after FDR correction..

Table S14. Differential expression of T regulatory genes between HG-*S. solidus* infected and control HR stickleback in week 6

ANOVA results	numDF	denDF	F-value	p-value	pseudo R2
il-16	1	4	2.7784351	0.1709	0.3274503
foxp3	1	4	12.615158	0.0238	0.677801
tgf-β	1	4	63.60417	**0.0013**	0.8974288

The statistical models were based on log10-transformed calibrated normalized relative quantities (CNRQ values). The weight of the fish was included as covariate to account for size related effects. Data from genes from significantly differentially expressed functional gene groups was analyzed with linear mixed models (LMMs; function lme() from *nlme*) and analyses of variance (ANOVAs). Conditional pseudo R^2 values (Nakagawa & Schielzeth, 2013; Johnson, 2014) were calculated with sem.model.fits() from *piecewiseSEM* (Lefcheck, 2015). Differentially expressed genes are marked in bold letters if significant after FDR correction.

Table S15. Differential expression of complement genes between HG-*S. solidus* infected and control (HR and LR) stickleback in week 9

ANOVA results	numDF	denDF	F-value	p-value	pseudo R2
cfb	1	10	10.051180	**0.0100**	0.7509158
c7	1	10	0.000858	0.9772	0.1542638
c9	1	10	5.861681	0.0360	0.9810338

The statistical models were based on log10-transformed calibrated normalized relative quantities (CNRQ values). The weight of the fish was included as covariate to account for size related effects. Data from genes from significantly differentially expressed functional gene groups was analyzed with linear mixed models (LMMs; function lme() from *nlme*) and analyses of variance (ANOVAs). Conditional pseudo R^2 values (Nakagawa & Schielzeth, 2013; Johnson, 2014) were calculated with sem.model.fits() from *piecewiseSEM* (Lefcheck, 2015). Differentially expressed genes are marked in bold letters if significant after FDR correction.

Table S16. Differential immune gene expression between *D. pseudospathaceum* infected and control stickleback

contrast						
P:H		Df	SumsOfSqs	F.Model	Pr(<F)	R2
CTRL - D:LR&HR	all genes (D effect)	1	3.913	1.53871	0.12119	0.02230
	all genes (H:T interaction)	2	11.820	2.32405	0.01040	0.06737

The statistical models were based on log10-transformed calibrated normalized relative quantities (CNRQ values). Non-parametric permutational multivariate analyses of variance (PERMANOVA) were calculated on Euclidean distances and 10,000 permutations that were constrained within fish family. The weight of the fish was included as covariate to account for size related effects. D effect: effect of *D. pseudospathaceum* infection. In this case, gene expression was only affected by an interaction between host type and time.

Table S17. Multivariate statistics (PERMANOVA results) of the effect of *S. solidus* – *D. pseudospathaceum* co-infection on stickleback immune gene expression

	contrast						
T	P:H		Df	SumsOfSqs	F.Model	Pr(<F)	R2
3	Co-LG - CTRL:LR&HR	all genes	1	3.830	1.39407	0.2198	0.05774
3	Co-HG - CTRL:LR&HR	all genes	1	6.807	1.88755	0.1499	0.07230
3	Co-HG – Co-LG:LR&HR	all genes	1	4.138	1.22793	0.5500	0.04170
6	Co-LG - CTRL:LR&HR	all genes	1	4.754	1.82810	0.1263	0.06671
6	Co-HG - CTRL:LR&HR	all genes	1	5.814	2.43653	0.05399	0.09334
6	Co-HG – Co-LG:LR&HR	all genes	1	3.696	1.3185	0.2642	0.04603
9	Co-LG - CTRL:LR&HR	all genes	1	4.926	2.02926	0.05299	0.08354
9	Co-HG - CTRL:LR&HR	all genes (P effect)	1	3.169	1.5420	0.1608	0.04346
		all genes (H effect)	1	10.510	5.1148	0.0216	0.14414
9	Co-HG – CTRL:HR	all genes	1	1.348	0.6052	0.6902	0.04097
9	Co-HG – CTRL:LR	all genes	1	6.6020	3.8846	0.0178	0.22315
		innate	1	5.1340	5.4308	**0.0195**	0.28195
		adaptive	1	1.1485	5.2576	**0.0122**	0.25374
		complement	1	0.3195	0.59627	0.4433	0.04664
		Th1	1	0.40863	4.8038	**0.0232**	0.26610
		Th2	1	0.42719	4.9585	**0.0226**	0.23610
		Treg	1	0.59940	11.6801	**0.0074**	0.47104
9	Co-HG – Co-LG:LR&HR	all genes	1	1.210	0.4679	0.7198	0.01348

The statistical models were based on log10-transformed calibrated normalized relative quantities (CNRQ values). The weight of the fish was included as covariate to account for size related effects. Non-parametric permutational multivariate analyses of variance (PERMANOVA) were calculated on Euclidean distances and 10,000 permutations that were constrained within fish family. PERMANOVA results were FDR corrected. If significant (marked in bold letters), single genes were analyzed with linear mixed models (LMMs). Statistics for differences between host types or interactions are mentioned whenever significant.

Table S18. Differential immune gene expression between HG-*S. solidus* - *D. pseudospathaceum* co-infected and control LR stickleback in week 9

ANOVA results	numDF	denDF	F-value	p-value	pseudo R2
marco	1	10	5.880190	0.0358	0.3029049
mst1ra	1	10	1.0458040	0.3306	0.7409858
mif1	1	10	8.678699	0.0146	0.6739742
il-1β	1	10	2793093.4	**< 0.0001**	0.9999997
tnfr1	1	10	5.668682	0.0386	0.5735934
saal1	1	10	1.5908441	0.2358	0.1020698
csf3r	1	10	4.343719	0.0638	0.2487352
p22^{phox}	1	10	4.312283	0.0646	0.4603152
nkef-β	1	10	7.287676	0.0223	0.2660523
sla1	1	10	5.184409	0.0460	0.2948069
cd97	1	10	5.056703	0.0483	0.286804
stat4	1	10	1.985945	0.1891	0.4252173
stat6	1	10	7.755325	0.0193	0.4208772
igm	1	10	5.580071	0.0398	0.7597721
cd83	1	10	0.1557763	0.7014	0.0529079
foxp3	1	10	17.383392	**0.0019**	0.7420727
tgf-β	1	10	10.890636	**0.0080**	0.4390618
il-16	1	10	10.284613	**0.0094**	0.4926989
mhcII	1	10	0.457965	0.5139	0.5255997
tcr-β	1	10	0.233701	0.6392	0.5590239

The statistical models were based on log10-transformed calibrated normalized relative quantities (CNRQ values). The weight of the fish was included as covariate to account for size related effects. Data from genes from significantly differentially expressed functional gene groups was analyzed with linear mixed models (LMMs; function lme() from *nlme*) and analyses of variance (ANOVAs). Conditional pseudo R^2 values (Nakagawa & Schielzeth, 2013; Johnson, 2014) were calculated with sem.model.fits() from *piecewiseSEM* (Lefcheck, 2015). Differentially expressed genes are marked in bold letters if significant after FDR correction.

Table S19. Differential immune gene expression between *S. solidus* infected and co-infected stickleback

T	contrast P:H		Df	SumsOfSqs	F.Model	Pr(<F)	R2
3	Co-LG - LG:LR&HR	all genes	1	1.979	0.96971	0.4699	0.04058
3	Co-HG - HG:LR&HR	all genes	1	2.275	0.63169	0.5374	0.02671
6	Co-LG - LG:LR&HR	all genes	1	3.354	1.3296	0.1487	0.04992
6	Co-HG - HG:LR&HR	all genes	1	3.566	1.37490	0.2674	0.07615
9	Co-LG -LG:LR&HR	all genes	1	1.156	0.46060	0.75532	0.02303
9	Co-HG -HG:LR&HR	all genes	1	18.471	6.4611	0.005899	0.18743
		innate	1	0.433	0.4466	0.60324	0.01343
		adaptive	1	0.1859	0.6555	0.56034	0.02125
		complement	1	17.852	11.1198	0.005799	0.31020
		Th1 (P effect)	1	0.02807	0.7082	0.4527	0.02336
		Th1 (H effect)	1	0.02855	0.7205	0.0268	0.02377
		Th2 (P:H interaction)	1	0.5031	3.2453	0.0208	0.10287
		Treg	1	0.05298	1.3689	0.26737	0.03918
9	Co-HG -HG:LR	*Th1*	1	0.03520	0.6851	0.45035	0.03320
		Th2	1	0.27530	3.5893	0.06289	0.13303
9	Co-HG -HG:HR	*Th1*	1	0.034140	3.3354	0.07979	0.26900
		Th2	1	0.24033	1.27777	0.1914	0.11415

The statistical models were based on log10-transformed calibrated normalized relative quantities (CNRQ values). The weight of the fish was included as covariate to account for size related effects. Non-parametric permutational multivariate analyses of variance (PERMANOVA) were calculated on Euclidean distances and 10,000 permutations that were constrained within fish family. Statistics for differences between host types or interactions are mentioned whenever significant. PERMANOVA results were FDR corrected. In this case, no result remained significant after FDR correction.

Table S20. Differential immune gene expression between *D. pseudospathaceum* infected and co-infected stickleback

T	contrast P:H		Df	SumsOfSqs	F.Model	Pr(<F)	R2
3	Co-LG - D:LR&HR	all genes	1	1.448	0.61862	0.6900	0.02419
3	Co-HG - D:LR&HR	all genes	1	4.478	1.32821	0.2193	0.05041
6	Co-LG - D:LR&HR	all genes	1	2.580	0.79285	0.65243	0.03015
6	Co-HG - D:LR&HR	all genes	1	6.998	2.29249	0.06219	0.08835
9	Co-LG -D:LR&HR	all genes	1	2.249	0.79498	0.4033	0.03304
9	Co-HG -D:LR&HR	all genes (P effect)	1	2.052	0.8567	0.56224	0.02515
9	Co-HG -D:LR&HR	all genes (H effect)	1	9.948	4.1524	0.04440	0.12191
9	Co-HG -D:LR	all genes	1	4.893	2.4794	0.03130	0.14528
		innate	1	1.4688	1.4344	0.1450	0.08567
		adaptive	1	1.8106	8.7102	0.03170	0.35095
		complement	1	1.6133	2.17597	0.06329	0.14185
		Th1	1	0.71763	12.6316	0.0354	0.47246
		Th2	1	0.91437	8.8065	0.0478	0.37766
		Treg	1	0.54614	11.2381	0.0329	0.43678
9	Co-HG -D:HR	all genes		2.940	1.00147	0.4807	0.07760

The statistical models were based on log10-transformed calibrated normalized relative quantities (CNRQ values). The weight of the fish was included as covariate to account for size related effects. Non-parametric permutational multivariate analyses of variance (PERMANOVA) were calculated on Euclidean distances and 10,000 permutations that were constrained within fish family. Statistics for differences between host types are mentioned when significant and infection effects where then tested for HR and LR host types separately. PERMANOVA results were FDR corrected. In this case, no results remained significant after FDR correction. D: *Diplostomum pseudospathaceum* infection.

References

Aerts-Toegaert, C., Heirman, C., Tuyaerts, S., Corthals, J., Aerts, J. L., Bonehill, A., … Breckpot, K. (2007). CD83 expression on dendritic cells and T cells: Correlation with effective immune responses. *European Journal of Immunology*, *37*(3), 686–695. doi:10.1002/eji.200636535

Bedard, K., & Krause, K.-H. (2007). The NOX Family of ROS-Generating NADPH Oxidases: Physiology and Pathophysiology. *Physiological Reviews*, *87*(1), 245–313. doi:10.1152/physrev.00044.2005

Birrer, S. C., Reusch, T. B. H., & Roth, O. (2012). Salinity change impairs pipefish immune defence. *Fish and Shellfish Immunology*, *33*(6), 1238–1248. doi:10.1016/j.fsi.2012.08.028

Brunner, F. S. (2016). *Parasitism along the speciation continuum - Variation of parasite effects across speciation stages of Gasterosteus aculeatus and their modification by environmental changes*. University of London. Retrieved from https://www.dora.lib4ri.ch/eawag/islandora/object/eawag:13601

Brunner, F. S., Anaya-Rojas, J. M., Matthews, B., & Eizaguirre, C. (2017). *Experimental evidence that parasites drive eco-evolutionary feedbacks*. *Proceedings of the National Academy of Sciences* (Vol. 114). doi:10.1073/pnas.1619147114

Calandra, T., & Roger, T. (2003). Macrophage migration inhibitory factor: a regulator of innate immunity. *Nature Reviews Immunology*, *3*(10), 791–800. doi:10.1038/nri1200

Haarder, S., Kania, P. W., Bahlool, Q. Z. M., & Buchmann, K. (2013). Expression of immune relevant genes in rainbow trout following exposure to live Anisakis simplex larvae. *Experimental Parasitology*, *135*(3), 564–569. doi:10.1016/j.exppara.2013.09.011

Haase, D., Rieger, J. K., Witten, A., Stoll, M., Bornberg-Bauer, E., Kalbe, M., & Reusch, T. B. H. (2014). Specific Gene Expression Responses to Parasite Genotypes Reveal Redundancy of Innate Immunity in Vertebrates. *PLoS ONE*, *9*(9), e108001. doi:10.1371/journal.pone.0108001

Hibbeler, S., Scharsack, J. P., & Becker, S. (2008). Housekeeping genes for quantitative expression studies in the three-spined stickleback Gasterosteus aculeatus. *BMC Molecular Biology*, *9*(1), 18. doi:10.1186/1471-2199-9-18

Huang, Y., Chain, F. J. J., Panchal, M., Eizaguirre, C., Kalbe, M., Lenz, T. L., … Feulner, P. G. D. (2016). Transcriptome profiling of immune tissues reveals habitat-specific gene expression between lake and river sticklebacks. *Molecular Ecology*, *25*(4), 943–958. doi:10.1111/mec.13520

Johnson, P. C. D. (2014). Extension of Nakagawa & Schielzeth's R2GLMM to random slopes models. *Methods in Ecology and Evolution*, *5*(9), 944–946. doi:10.1111/2041-210X.12225

Kaplan, M. H. (2005). STAT4: A critical regulator of inflammation in vivo. *Immunologic Research*, *31*(3), 231–241. doi:10.1385/IR:31:3:231

Kasheta, M., Painter, C. A., Moore, F. E., Lobbardi, R., Bryll, A., Freiman, E., … Ceol, C. J. (2017). Identification and characterization of T reg–like cells in zebrafish. *The Journal of Experimental Medicine*, *214*(12), 3519–3530. doi:10.1084/jem.20162084

Kissick, H. T., Dunn, L. K., Ghosh, S., Nechama, M., Kobzik, L., & Arredouani, M. S. (2014). The scavenger receptor MARCO modulates TLR-induced responses in dendritic cells. *PLoS ONE*, *9*(8), 1–11. doi:10.1371/journal.pone.0104148

Kovacevic, N., Hagen, M. O., Xie, J., & Belosevic, M. (2015). The analysis of the acute phase response during the course of Trypanosoma carassii infection in the goldfish (Carassius auratus L.). *Developmental and Comparative Immunology*, *53*(1), 112–122. doi:10.1016/j.dci.2015.06.009

Kraal, G., Van Der Laan, L. J. W., Elomaa, O., & Tryggvason, K. (2000). The macrophage receptor MARCO. *Microbes and Infection*, *2*(3), 313–316. doi:10.1016/S1286-4579(00)00296-3

Leemans, J. C., te Velde, A. A., Florquin, S., Bennink, R. J., de Bruin, K., van Lier, R. A. W., … Hamann, J. (2004). The Epidermal Growth Factor-Seven Transmembrane (EGF-TM7) Receptor CD97 Is Required for Neutrophil Migration and Host Defense. *The Journal of Immunology*, *172*(2), 1125–1131. doi:10.4049/jimmunol.172.2.1125

Lefcheck, J. S. (2015). piecewiseSEM: Piecewise structural equation modeling in R for ecology, evolution, and systematics. *Methods in Ecology and Evolution*, *7*(5), 573–579. doi:10.1111/2041-210X.12512

Lenz, T. L., Eizaguirre, C., Becker, S., & Reusch, T. B. H. (2009). RSCA genotyping of MHC for high-throughput evolutionary studies in the model organism three-spined stickleback Gasterosteus aculeatus. *BMC Evolutionary Biology*, *9*, 57. doi:10.1186/1471-2148-9-57

Marton, N., Baricza, E., Érsek, B., Buzás, E. I., & Nagy, G. (2015). The emerging and diverse roles of Src-like adaptor proteins in health and disease. *Mediators of Inflammation*, *2015*. doi:10.1155/2015/952536
Maxson, J. E., Gotlib, J., Pollyea, D. A., Fleischman, A. G., Agarwal, A., Eide, C. A., … Tyner, J. W. (2013). Oncogenic CSF3R Mutations in Chronic Neutrophilic Leukemia and Atypical CML. *New England Journal of Medicine*, *368*(19), 1781–1790. doi:10.1056/NEJMoa1214514
Mayumi, M., Takeda, Y., Hoshiko, M., Serada, K., Murata, M., Moritomo, T., … Sumimoto, H. (2008). Characterization of teleost phagocyte NADPH oxidase: Molecular cloning and expression analysis of carp (Cyprinus carpio) phagocyte NADPH oxidase. *Molecular Immunology*, *45*(6), 1720–1731. doi:10.1016/j.molimm.2007.09.028
Nakagawa, S., & Schielzeth, H. (2013). A general and simple method for obtaining R2 from generalized linear mixed-effects models. *Methods in Ecology and Evolution*, *4*(2), 133–142. doi:10.1111/j.2041-210x.2012.00261.x
Premachandra, H. K. A., Elvitigala, D. A. S., Bathige, S. D. N. K., Whang, I., Lee, Y., De Zoysa, M., & Lee, J. (2013). Genomic structure and immunological response of an STAT4 family member from rock bream (Oplegnathus fasciatus). *Fish and Shellfish Immunology*, *35*(6), 1829–1837. doi:10.1016/j.fsi.2013.09.011
Rao, D. N., & Naqvi, R. A. (2011). FoxP3: A key player in T regulatory biology. *Indian Journal of Clinical Biochemistry*, *26*(1), 1–2. doi:10.1007/s12291-011-0112-8
Rhodes, L. D., Wallis, S., & Demlow, S. E. (2009). Genes associated with an effective host response by Chinook salmon to Renibacterium salmoninarum. *Developmental and Comparative Immunology*, *33*(2), 176–186. doi:10.1016/j.dci.2008.08.006
Robertson, S., Bradley, J. E., & MacColl, A. D. C. (2015). Measuring the immune system of the three-spined stickleback - investigating natural variation by quantifying immune expression in the laboratory and the wild. *Molecular Ecology Resources*, *16*(3), 701–713. doi:10.1111/1755-0998.12497
Rønneseth, A., Ghebretnsae, D. B., Wergeland, H. I., & Haugland, G. T. (2015). Functional characterization of IgM^+ B cells and adaptive immunity in lumpfish (Cyclopterus lumpus L.). *Developmental and Comparative Immunology*, *52*(2), 132–143. doi:10.1016/j.dci.2015.05.010
Shau, H., Gupta, R. K., & Golub, S. H. (1993). Identification of a natural killer enhancing factor (NKEF) from human erythroid cells. *Cellular Immunology*. doi:10.1006/cimm.1993.1043
Smith-Garvin, J. E., Koretzky, G. A., & Jordan, M. S. (2009). T Cell Activation. *Annual Review of Immunology*, *27*(1), 591–619. doi:10.1146/annurev.immunol.021908.132706
Stutz, W. E., Schmerer, M., Coates, J. L., & Bolnick, D. I. (2015). Among-lake reciprocal transplants induce convergent expression of immune genes in threespine stickleback. *Molecular Ecology*, *24*(18), 4629–4646. doi:10.1111/mec.13295
Tabbara, I. A. (1993). Granulocyte colony-stimulating factor. *Southern Medical Journal*, *86*(3), 350–5. Retrieved from http://www.ncbi.nlm.nih.gov/pubmed/7680827
Wang, M. H., Zhou, Y. Q., & Chen, Y. Q. (2002). Macrophage-stimulating protein and RON receptor tyrosine kinase: Potential regulators of macrophage inflammatory activities. *Scandinavian Journal of Immunology*, *56*(6), 545–553. doi:10.1046/j.1365-3083.2002.01177.x
Wang, T., & Secombes, C. J. (2013). The cytokine networks of adaptive immunity in fish. *Fish and Shellfish Immunology*, *35*(6), 1703–1718. doi:10.1016/j.fsi.2013.08.030
Wen, Y., Shao, J. Z., Xiang, L. X., & Fang, W. (2006). Cloning, characterization and expression analysis of two Tetraodon nigroviridis interleukin-16 isoform genes. *Comparative Biochemistry and Physiology - B Biochemistry and Molecular Biology*, *144*(2), 159–166. doi:10.1016/j.cbpb.2006.02.012
Yanagi, Y., Yoshikai, Y., Leggett, K., Clark, S. P., Aleksander, I., & Mak, T. W. (1984). A human T cell-specific cDNA clone encodes a protein having extensive homology to immunoglobulin chains. *Nature*, *308*(5955), 145–9. doi:10.1038/308145a0
Zhu, L., Nie, L., Zhu, G., Xiang, L., & Shao, J. (2013). Advances in research of fish immune-relevant genes: a comparative overview of innate and adaptive immunity in teleosts. *Developmental and Comparative Immunology*, *39*(1–2), 39–62. doi:10.1016/j.dci.2012.04.001
